# Resistance to Arsenite and Arsenate in *Saccharomyces cerevisiae* Arises through the Subtelomeric Expansion of a Cluster of Yeast Genes

**DOI:** 10.3390/ijerph19138119

**Published:** 2022-07-01

**Authors:** Irene Stefanini, Monica Di Paola, Gianni Liti, Andrea Marranci, Federico Sebastiani, Enrico Casalone, Duccio Cavalieri

**Affiliations:** 1 Department of Life Sciences and Systems Biology, University of Turin, 10123 Turin, Italy; irene.stefanini@unito.it; 2 Department of Biology, University of Florence, Sesto Fiorentino, 50019 Florence, Italy; monica.dipaola@unifi.it (M.D.P.); enrico.casalone@unifi.it (E.C.); 3 National Centre for Scientific Research (CNRS), National Institute of Health and Medical Research (INSERM), Institute for Research on Cancer and Aging (IRCAN), Université Côte d’Azur, 06103 Nice, France; gianni.liti@unice.fr; 4 Core Research Laboratory, Oncogenomics Unit, Istituto di Fisiologia Clinica, Institute for Cancer Research and Pre-vention (ISPRO), 56124 Pisa, Italy; andrea.marranci@gmail.com; 5 Institute for Sustainable Plant Protection, National Research Council (IPSP-CNR), Sesto Fiorentino, 50019 Florence, Italy; federico.sebastiani@ipsp.cnr.it

**Keywords:** arsenic, resistance, *Saccharomyces cerevisiae*, *ARR1*, *ARR2*, *ARR3*, duplicated DNA, translocated DNA

## Abstract

Arsenic is one of the most prevalent toxic elements in the environment, and its toxicity affects every organism. Arsenic resistance has mainly been observed in microorganisms, and, in bacteria, it has been associated with the presence of the Ars operon. In *Saccharomyces cerevisiae*, three genes confer arsenic resistance: *ARR1*, *ARR2*, and *ARR3*. Unlike bacteria, in which the presence of the Ars genes confers per se resistance to arsenic, most of the *S. cerevisiae* isolates present the three *ARR* genes, regardless of whether the strain is resistant or sensitive to arsenic. To assess the genetic features that make natural *S. cerevisiae* strains resistant to arsenic, we used a combination of comparative genomic hybridization, whole-genome sequencing, and transcriptomics profiling with microarray analyses. We observed that both the presence and the genomic location of multiple copies of the whole cluster of *ARR* genes were central to the escape from subtelomeric silencing and the acquisition of resistance to arsenic. As a result of the repositioning, the *ARR* genes were expressed even in the absence of arsenic. In addition to their relevance in improving our understanding of the mechanism of arsenic resistance in yeast, these results provide evidence for a new cluster of functionally related genes that are independently duplicated and translocated.

## 1. Introduction

Arsenic is one of the most prevalent toxic elements in the environment, and it is usually present in low amounts in rocks, soil, and water [1]. Human activities contribute to the mobilization of arsenic through mining and mineral processing, as well as its use in pesticides, herbicides, and wood preservatives [2]. In 2010, the WHO reported that groundwater in Argentina, Chile, China, India (West Bengal), Mexico, the United States of America, and Bangladesh contained high and alarming levels of inorganic arsenic [3]. Prolonged exposure to soluble inorganic arsenic can lead to chronic poisoning-induced effects, including peripheral neuropathy, diabetes, cardiovascular diseases, and cancer [3]. In nature, arsenic is present in various forms, but mainly the trivalent and pentavalent forms, which can exist both in organic and inorganic compounds [4]. The type of toxicity induced by arsenic compounds depends on the type of arsenic and on the type of affected cells [5]. The organic form of arsenic is, in general, less toxic than its inorganic form [4,5]. In addition, trivalent arsenic (also referred to here as arsenite) is more toxic than pentavalent arsenic (arsenate) because of enhanced cellular uptake, greater intracellular accumulation, and a higher affinity for protein thiols and sulfhydryl groups [5]. By binding the SH groups of proteins, trivalent arsenic can alter their structure, thereby inducing various deleterious effects on the cell [5]. In addition, trivalent arsenic induces the formation of reactive oxygen species (ROS) by unknown mechanisms and DNA methylation by either interacting with DNA methylating enzymes or by depleting S-adenosylmethionine, the donor of the methyl group [6]. Both the trivalent and pentavalent forms of arsenic can enter the cell through channels and transporters. Aquaporin channels and glucose transporters can import As^3+^, while pentavalent inorganic arsenic, being an analog of inorganic phosphate, enters the cell via the phosphate transporter [5], thus affecting phosphate-dependent cellular processes.

Mechanisms for resistance to arsenic have been found in several organisms. Resistance is mainly due to a reduction in the cellular uptake of arsenic as the result of mutations determining conformational changes in the proteins involved in the uptake and/or activation of efflux systems. In bacteria, resistance is achieved due to five genes organized in operons and located in either chromosomes or plasmids. Such genes encode for two transcriptional regulators (ArsR and ArsD) [7]; an arsenate reductase (ArsC), which converts arsenate to arsenite, a more easily extrudable form of As [8]; and the proteins ArsA and ArsB, which compose an arsenical ATPase [9] that extrudes arsenite from the cell. The arsenic resistance operon has been found in several bacterial species, including *Bacillus subtilis* [10], *Pseudomonas aeruginosa* [11], *Staphylococcus aureus* [12], and *Staphylococcus xylosus* [13]. Once arsenic enters the cell, ArsR and ArsD are activated and in turn induce the expression of the other genes of the operon [7,14]. In Eukaryota, specific arsenical resistance genes are rarely found, and the tolerance to arsenic may be achieved through the activity of non-specific multidrug resistance proteins [15]. However, in the budding yeast *Saccharomyces cerevisiae*, three contiguous genes, named arsenical resistance (ARR) genes, have been identified as responsible for its resistance to arsenic [15]. The three genes encode for a transcription activator (*ARR1*), which is activated by the binding of trivalent arsenic [16]; an arsenate reductase (*ARR2*), which converts arsenate to arsenite [17]; and a plasma membrane metalloid/H+ antiporter (*ARR3*) [18]. As a concise comparison, the yeast Arr1p resembles the bacterial ArsR and ArsD, Arr2p corresponds to bacterial ArsC and could promote resistance against arsenic by reducing the pentavalent form into the trivalent form—as also observed in the *S. cerevisiae* resistance mechanisms to other heavy metals such as chromium [19]—and Arr3p acts similarly to the bacterial ArsB–ArsA complex. Despite the similarities among yeast and bacterial arsenic resistance genes, major differences are present. The most important difference is that while in bacteria the presence of the operon confers the resistance to the cell, the presence of the *ARR* genes in yeast is not sufficient to induce resistance. Every *S. cerevisiae* strain characterized thus far presents the three *ARR* genes, regardless of whether the strain is resistant or sensitive to arsenic. Hence, there must be additional factors in yeast regulating the efficacy of the *ARR* genes. Here, to try to fill this knowledge gap, we used high-throughput analyses encompassing comparative genomic hybridization (CGH), whole-genome sequencing, and transcriptomics profiling with microarray to identify the genetic features that make natural *S. cerevisiae* strains resistant to arsenic.

## 2. Materials and Methods

### 2.1. Yeast Strains and Growth Conditions

A list of *S. cerevisiae* strains used in this study is reported in Table S1. Yeast strains were grown at 30 °C in YPD (1% yeast extract, 2% peptone, and 2% glucose), with the possible addition of 2% agar to obtain a solid medium. The effects of sodium arsenite and arsenate were evaluated using overnight broth cultures by spotting 10 µL of sterile water suspensions containing 10^2^, 10^3^, and 10^4^ cells onto YPD supplemented with various concentrations of one of the two As salts (1.25 mM, 2.5 mM, 5 mM, 10 mM, and 15 mM for sodium arsenite and 6.25 mM, 12.5 mM, 25 mM, 50 mM, and 75 mM for sodium arsenate). Plates were incubated at 30 °C and the strains’ growth was measured after three days incubation, compared to a control treatment (YPD), and scored as 0 (no growth), + (growth), or ++ (growth as in control). Tests were carried out in two independent biological replicates.

### 2.2. Comparative Genomic Hybridization (CGH)

Microarray slides for CGH were fabricated in the Bauer Center for Genomics Research at Harvard University (http://sysbio.harvard.edu/csb/ (accessed on 1 December 2021)). The slides were constructed using the *S. cerevisiae* Genome Oligo Set (Operon Technologies, Alameda, CA, USA) composed of 6240 optimized oligonucleotides (70 mers), each representing one yeast gene. We followed the same procedure applied to the transcriptional microarray data for the normalization of CGH. CGH microarray data, raw and normalized, were submitted to Gene Expression Omnibus (GEO), under the National Center for Biotechnology Information (NCBI) accession number GSE136466 (detailed accession numbers in Table S4). We used BY4743, a diploid derivative of S288c, as a reference strain for the hybridization. For these experiments, overnight cultures of selected yeast strains in YPD were harvested, and cellular pellets were conserved at −20 °C. Genomic DNA was then extracted from each pellet using the phenol-chloroform method [20]. The extracted genomic DNA was concentrated using a Microcon YM-30 filter (Millipore, Burlington, MA, USA) and fragmented by sonication. DNA labeling and microarray hybridization were performed as previously described in [21]. Fluorescent DNA bound to the microarray was detected with a GenePix 4000B microarray scanner (Axon Instruments, Union City, CA, USA) using the GenePixPro6.1 software package to quantify the microarray fluorescence. The control microarray spot quality required the following features: 50% as the minimum percentage of pixels for which the foreground intensity was greater than the background intensity +2 standard deviation values; 80 pixels as the minimum number of pixels; and an absence of saturated pixels. Hybridizations were carried out in two independent technical replicates.

### 2.3. Strain Genome Sequencing, Analysis, and Assembly

The genomes of selected strains (SGU90 and SG60, which were As resistant, and 1014 and EM93, which were As sensitive) were extracted using the phenol-chloroform method. Whole-genome sequencing was performed using the Genome Analyser IIx (GAIIx) platform and HiSeq2000 sequencing instruments. The standard Illumina protocol with minor modifications was followed for the creation of short-insert paired-end libraries (Illumina Inc., Cat. # PE-930-1001). In brief, 2.0 µg of genomic DNA was sheared on a Covaris™ E220. The fragmented DNA was end repaired, adenylated, and ligated to Illumina-specific paired-end adaptors. To obtain a library of a very precise insert size (500 ± 25 bp), the DNA with adaptor-modified ends was size selected and purified using an E-gel agarose electrophoresis system (Invitrogen, Waltham, MA, USA). After size selection, the library was PCR amplified using 10 PCR cycles. A second library was obtained using the same protocol with the addition of, before adaptor ligation, a final step at 72 °C and a rapid cooling down to 4 °C. Each library was run in a fraction of a GAIIx flow cell lane in paired end mode with a 2 × 151 bp read length using a Sequencing kit v4 or on HiSeq2000 with a 2 × 101 bp read length using a TruSeq SBS Kit v3, both according to the standard Illumina operation procedures (Illumina, Inc., San Diego, CA, USA). Primary data analysis was carried out with the standard Illumina pipeline. The purity of the signal from each cluster was examined over the first 25 cycles and chastity = Highest_Intensity/(Highest_Intensity + Next_Highest_Intensity) was calculated for each cycle. To remove the least reliable data from the analysis, the reads were filtered according to chastity > 0.6 for all but one of the first 25 bases. If there were two bases, the read was subsequently removed. The Illumina reads were subjected to quality control (filtering and trimming) using the NGS QC Toolkit v2.3.3 [22]. Paired reads were filtered with the parameters −l 70, (cutOffReadLen4HQ) and −s 20 (cutOffQualScore), allowing for the elimination of reads with a PHRED quality score lower than 20 for more than 30% of their lengths. Moreover, the reads were trimmed at the 3′ end for bases with a PHRED quality score lower than 30. Paired reads were mapped to the reference genome (*Saccharomyces cerevisiae* S288c, NCBI ID = 559292) using bowtie2 version 2.2.6 [23]. The Illumina run generated on average 12.8 ± 6.6 million filtered reads and 12.4 ± 6.2 million mapped reads. Read coverage depth was calculated for each sequencing with the samtools depth function [24]. SNPs and indels were identified with the GATK analysis pipeline [25]. SNPs associated with arsenic resistance were identified as the SNPs present only in resistant strains by using a lab-made Python script to merge and compare vcf files. The identified SNPs were annotated using the SnpEff software [26]. Genomes were assembled using ABySS version 2.0 [27]. For each sequenced strain, ABySS was run testing k = 50, 58, 64, 74, 82, and 90; then, the best assembly was identified using AbySSExplorer [28]. Genes were predicted in the obtained unitigs using the Augustus algorithm [29], and the predicted genes were annotated through a BLAST search against the S288C nucleotide collection [30]. Raw, assembly, and annotation data were submitted to NCBI (details in Table S4). The Whole Genome Shotgun projects, encompassing the annotated genomes, were deposited in GenBank under the accession numbers WNEH00000000 (1014), WMLL00000000 (SG60), WNEF00000000 (SGU90), and WOFK00000000 (EM93).

### 2.4. Identification of Potential Duplications through Whole-Genome Sequencing

The depth of coverage was calculated for each nucleotide of the reference strain genome with the samtools depth function [24] and was normalized within the sample by dividing each value by the geometric mean of the depth of coverage for the corresponding sequencing. Then, the average depth was calculated in non-overlapping windows (1kbp) and log2 transformed. The resulting values were analyzed with the circular binary segmentation (CBS) algorithm using the DNAcopy R package [31] to identify the exact coordinates and length of the duplicated region in chromosome XVI encompassing the *ARR1*, *ARR2*, and *ARR3* genes. The reference sequence encompassing the *ARR* genes, and the region identified through CBS analysis, was extracted from the whole-genome sequence of the reference strain (GCF_000146045.2_R64_genomic downloaded from NCBI July 2019). We will refer to this region as the “*ARR* cluster”. Illumina reads from each strain were aligned against the *ARR* cluster sequence, and the unmapped read mates of the reads mapped against this sequence were selected by using the samtools view method and selecting (-f) the reads flagged as ‘5’ and removing (-F) the reads flagged as ‘77’ or ‘141’. Then, the selected reads were aligned against the whole-genome sequence of the reference strain using bowtie2 [23], and the mapped reads were filtered according to the mapping quality (-q 30) with the samtools view method. Depth coverage was calculated for each mapped nucleotide using the samtools depth method [24] and was inter-sequence (inter-strain) normalized by dividing the depth coverage value by the average depth coverage of the corresponding chromosome. 

### 2.5. Pulsed-Field Gel Electrophoresis and Southern Blotting Hybridization 

PFGE was carried out as described by Hage and Houseley [32]. Briefly, strains were grown overnight in YPD at 30 °C with shaking. The cells were washed with PFGE wash buffer (10 mM Tris pH 7.6, 50 mM EDTA); then, plug molds were prepared by mixing 0.25 × 108 yeast cells suspended in 50 μL PFGE wash buffer with lyticase (170 U/mL final concentration) and 50 μL of low-melting-point 0.4% agarose. The solidified plugs were incubated at 37 °C in wash buffer supplemented with 340 U/mL lyticase. After 1 h, the plugs were treated overnight at 50 °C with proteinase K (1 mg/mL). The following day, the plugs were washed four times for 30 min with PFGE wash buffer and stored at 4 °C in the same wash buffer. The gel for electrophoresis was prepared with 1% agarose in 0.5× TBE, and the CHEF system was programmed as follows: 24 h run, switch times (ramped) 60–120, and 6 V/cm voltage. After the run, the gel was stained with 10 μg/mL ethidium bromide. A Southern blot was then carried out in standard conditions to transfer the DNA to a nitrocellulose membrane (transfer time of at least 24 h). For Southern blotting hybridization, *ARR1* and *ARR3* probes were prepared as described in [33]. The primers used to prepare the probes by PCR were ARR1FW (5′-GAGAGGAACATGCCTTCTG-3′), ARR1REV (5′-GCTGCCGCTGTTTCTATTG-3′), ARR3FW (5′-GGTCATCCCAATCTAATGGG-3′), and ARR3REV (5′-GGAAATAGCAATTGCCAGGG-3′).

### 2.6. Transcriptional Analysis

Strains were grown in YPD (laboratory conditions) or synthetic wine must (SWM; simulating fermentation conditions). SWM contains 0.17% yeast nitrogen base without amino acids and (NH4)SO4, 0.15% casamino acids, 0.05% NH4Cl, 0.60% DL-malic acid, 0.02% citric acid, 0.15% L-tartaric acid, 21% glucose, and 0.20% anaerobic factors ergosterol–Tween 80 (total 10 mg/L ergosterol and 0.5 mL/L Tween 80). The pH was adjusted to 3.3 with KOH. All cells were grown at 28 °C. For YPD, the cells were grown aerobically in a shaker at 200 rpm and harvested at an optical density (OD) of 0.8 (A600). For SWM, the strains were grown aerobically overnight in SWM and were then diluted to 0.2 OD in SWM and left to grow to 0.8 OD in a shaker at 50 rpm. Anaerobic conditions were established by locked fermentation flasks with CO_2_ outlets filled with water. For all conditions, the cells were harvested by centrifugation (3000× *g* for 10 min) at then flash frozen using liquid nitrogen. The hybridization protocol is described in Townsend et al. [34]. The hybridization spots were visually inspected, and flawed spots were flagged (negative values). Raw and normalized data were submitted to Gene Expression Omnibus (GEO) under the National Center for Biotechnology Information (NCBI) accession number GSE3021.

## 3. Results

### 3.1. Resistance to Arsenite and Arsenate in Natural Saccharomyces cerevisiae Strains

The *S. cerevisiae* strains tested in this study were laboratory strains (EM93 [35], SK1 [36], W303 [37], BY4742, and BY4741 [38]) and environmental isolates from vineyard-related specimens in Tuscany, Italy (1014, SG60, SGU89, SGU90, SGU114, SGU406, SGU407 [39], M12, M28, and M57 [40]) (Table S1). A survey of their phenotypic characteristics revealed that some of these strains were highly resistant to arsenic; this was assessed by growing them in the presence of either trivalent arsenic (As^3+^, sodium arsenite, NaAsO_2_) or pentavalent arsenic (As^5+^, sodium arsenate, Na_2_HAsO_4_). While the laboratory strains and most of the isolates from the vineyard-related specimens were sensitive to 1.25 mM arsenite, SG60, SGU89, SGU90, and SGU114 were capable of growing on media supplemented with up to 5 mM arsenite (Table 1). These same strains also grew in the presence of up to 6.25 mM sodium arsenate; SG60 and SGU90 were shown to grow at up to a 12.5 mM concentration of this salt (Table 1). However, at a higher cell density, SG60, SGU89, SGU90, and SGU114 also grew in the presence of up to 25 mM arsenate (Table S2).

### 3.2. Comparative Genomic Hybridization (CGH) Reveals the Presence of Multiple Copies of the ARR1, ARR2, and ARR3 Genes in Arsenic-Resistant Strains

**Figure 1:**
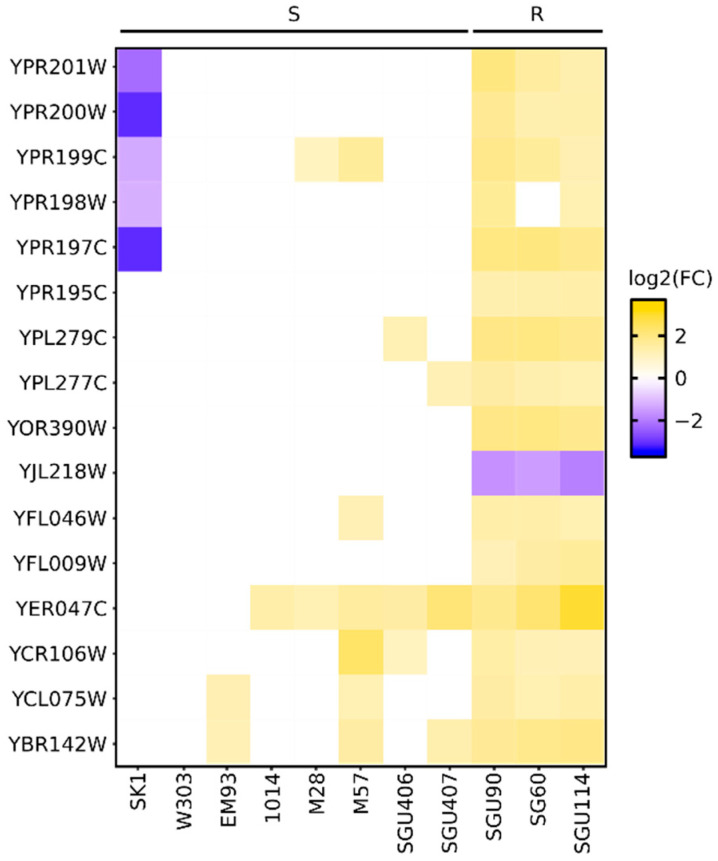
Heatmap of genes differentially represented in resistant and sensitive strains. Only genes differentially represented in arsenic-resistant strains and not differentially represented in sensitive strains, having an opposite logarithm with a two-fold or higher fold change (log2FC) in every sensitive strain or having the same log2FC in at maximum two sensitive strains, are shown. R—strains resistant to arsenic; S—strains sensitive to arsenic.

To assess whether the *S. cerevisiae* strains resistant to arsenic showed characteristic genomic settings, we evaluated the presence of relevant copy number variations (CNVs) in the arsenic-resistant strains SG60, SGU90, and SGU114 compared to the arsenic-sensitive strains SK1, W303, EM93, 1014, M28, M57, SGU406, and SGU407. With this aim, we used comparative genomic hybridization (CGH) to obtain lists of genes that were differentially represented in the tested strains compared to the diploid laboratory strain BY4743 (Table S3). With this approach, we identified 23 genes that were differentially represented (Wilcoxon test, false discovery rate (FDR) adjusted *p*-value < 0.05) in every arsenic-resistant strain and not differentially represented in the majority (more than 75%) of sensitive strains (Figure 1). Of the seven genes that were under-represented in the arsenic-resistant strains, three were either pseudogenes or dubious ORFs (YAR053W, YAR060C, and YAR061W), while the other four genes encoded proteins of unknown function (YAL064W-B, YER188W, YFL068W, and YLR462W) (Figure 1). Sixteen genes were over-represented in the arsenic-resistant strains (Figure 1); among them were the three genes known to be involved in the mechanism of resistance to arsenic, *ARR1* (YPR199C), *ARR2* (YPR200W), and *ARR3* (YPR201W), which code for a transcriptional activator, an arsenate reductase, and a metalloid/H+ antiport, respectively. Other genes that were over-represented in the arsenic-resistant strains code for a maltose permease (*MAL31*-YBR298C), an alcohol dehydrogenase (*ADH7*-YCR105W), a transmembrane nucleoporin (POM33-YLL023C), proteins involved in fluoride export (*FEX1*-YOR390W and *FEX2*-YPL279C), a transcription factor (*RDS1*-YCR106W), a protein required for the progression of the cell cycle (*CDC4*-YFL009W), and a protein controlling telomere length (*RIF2*-YLR453C). Furthermore, two additional genes (YPR195C and YPR197C) with unknown functions, located in the same region of chromosome XVI where the *ARR* genes are located, were over-represented in the arsenic-resistant strains (Figure 1). This observation suggests a duplication of the terminal portion of the right arm of chromosome XVI and the association of this event with the strains’ resistance to arsenic. It should be noted that the gene *ARR1* (YPR199C) was over-represented in two strains that were sensitive to arsenic (M28 and M57).

**Figure 2:**
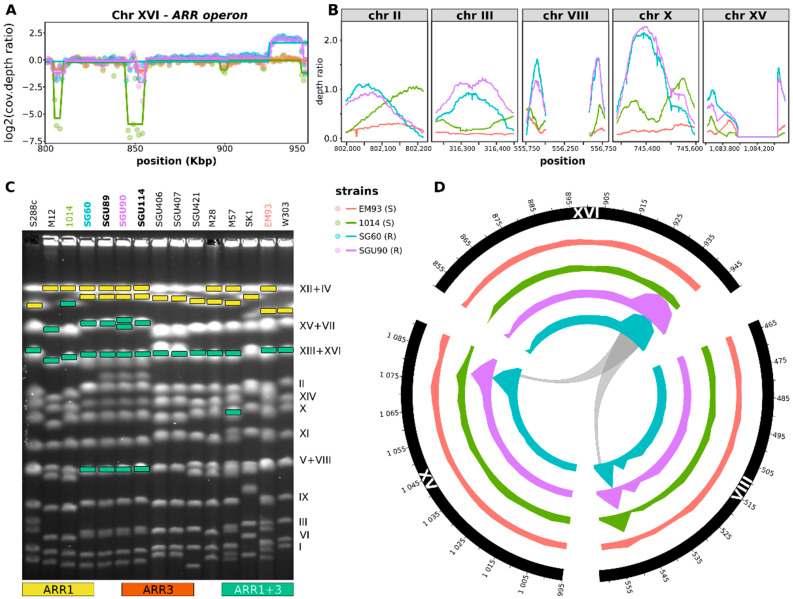
Identification of the duplicated region and its new genomic positions. (**A**) Results of CBS analysis highlighting the region of chromosome XVI encompassing the *ARR* genes. Points show the values of the ratio between the depth coverage in non-overlapping 1000 nt windows and the average depth coverage. Lines show the segment depth of coverage mean calculated with the CBS analysis. (**B**) Potential genomic region recipients of the additional copies of the *ARR* cluster. (**C**) Pulsed-field gel electrophoresis (PFGE) and *ARR1* or *ARR3* hybridization results confirming the new location of the *ARR* gene cluster in strains resistant to arsenic. After the PFGE run, DNA was transferred through Southern blot and hybridized with *ARR1*- or *ARR3*-labeled probes. Colored rectangles represent the bands identified through hybridization with *ARR1*, *ARR3*, or both genes, as indicated in the legend at the bottom of the figure. The tested strain names are listed on top of the figure, with strains resistant to arsenic written in bold. (**D**) Circos plot showing the regions potentially including additional copies in the strains resistant to arsenic and the corresponding depth of coverage for each sequenced strain. Coordinates are reported as Kbp. In the ‘strains’ legend, S and R stand for sensitive and resistant to arsenic, respectively.

To identify the possible locations of the additional copies of this region, we took advantage of the features of paired-end sequencing (Table S4). In fact, we searched for the reads mapped against the region of interest in the reference genome with read mates mapped against a distant position in the genome, hence flagged as unpaired by bowtie2 (Figure S1, Table S5). The read mates should align against the genomic region where the additional copy of the *ARR* gene cluster is located (Table S6). This technique has been widely used to detect transposable elements from whole-genome sequencing data [41]. Briefly, we obtained the sequence of the *ARR* cluster from the genome sequence of the reference strain S288C, selected the mapped reads for each sequenced strain, and found the corresponding read mates not mapped against the ARR cluster sequence. We then aligned the read mates against the whole genome of the reference strain and searched for genomic regions characterized by a depth of coverage similar to the whole-genome average depth of coverage (Figure S1). Using this approach, we were able to identify several genomic regions potentially including the additional copies of the *ARR* cluster (Figure S2). We further filtered these candidate recipient regions to consider only those longer than 100 bp, as shorter regions are unlikely to be confidently targeted by 150 bp Illumina reads (Figure 2B). In this way, we assessed that good candidate loci for the inclusion of the additional copies of the *ARR* cluster were on chromosomes II, III, VIII, X, and XV (Figure 2B and Table S7). To further investigate the position of these insertion loci, we assembled the genomes of the sequenced strains using the ABySS de novo genome assembler and searched for the unitigs that partially matched with the *ARR* cluster sequence (Table S8). We were able to reconstruct a large part of the genomes (min: 11.18 Mbp over a total of 12.16 Mbp of the reference strain genome) with a unitig N50 ranging from a minimum of 8270 bp (strain 1014) to a maximum of 28,372 bp (strain SG60) (Table S8). Through Augustus analysis, we were able to predict between 5317 (for strain 1014) and 5916 (for strain SG60) potential genes, among which some were not present in the reference S288C but were present in other sequenced *S. cerevisiae* strains (Table S8). Among the contigs generated through genome assembly, we searched for those with a sequence matching in part the sequence of the *ARR* cluster and in part other locations in the genome of the reference strain (Table S8 and Supplementary Results). While we could not find contigs supporting the integration of the *ARR* cluster in chromosomes II, III, and X, our results indicated the precise location in chromosomes VIII and XV, starting from nucleotide 556,053 and from nucleotide 1,067,195, respectively (Supplementary Results). To validate the new position predicted with Illumina sequencing, pulsed-field gel electrophoresis (PFGE) was carried out followed by Southern blot and DNA hybridization with *ARR1* or *ARR3* probes (Figure 2C). Using this approach, we were able to confirm that the strains resistant to arsenic carried additional *ARR1* and *ARR3* copies in chromosomes XV/VII and V/VIII. It should be noted that the PFGE and Southern blot/hybridization analyses suggested that every tested strain (sensitive or resistant to arsenic) bore additional copies of the *ARR1* and *ARR3* genes in chromosomes XII/IV (Figure 2C). However, neither the CGH microarray analysis (Figure 1) nor the CNV analysis carried out on sequencing data (Figure 2A) indicated the presence of multiple copies of these genes on chromosomes XII or IV in arsenic-sensitive strains. Thus, the bands corresponding to chromosomes XII/IV could be ascribed either to aspecific hybridization or to a lack of resolution of the subtelomeric regions, hindering the confirmation of the results. On the other hand, the hybridization bands corresponding to chromosomes XV/VII and V/VIII in resistant strains only (Figure 2C) further support the presence of additional *ARR* gene cluster copies on chromosomes XV and VIII (Figure 2D).

### 3.3. Transcriptomic Profiling Highlights Patterns Associated with the Genetic Makeup of Arsenic-Resistant Strains

**Figure 3:**
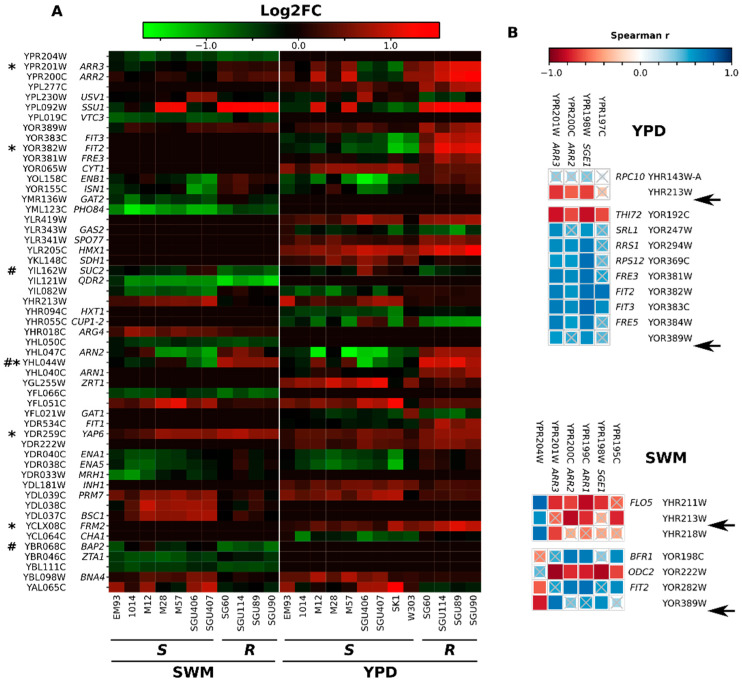
Transcriptomic analysis on strains resistant and sensitive to arsenic. (**A**) Heatmap reporting the expression levels of genes expressed at significantly different levels among resistant and sensitive strains. * and # represent genes differentially expressed in opposite directions (over vs. underexpressed and vice versa) in resistant compared to sensitive strains in YPD and SWM, respectively. (**B**) Spearman correlation analysis among expression levels of DEGs located in the *ARR* cluster and in the genomic regions, including the additional copies of the cluster (genes with names starting with “YH” are located in chromosome VIII, while those starting with “YO” are located in chromosome XV). For each comparison, the size and color of the squares indicate the calculated Spearman r. Crossed squares indicate not significantly correlated couples (fdr > 0.05). Black arrows indicate the position of the insertion of the additional copy of the *ARR* gene cluster in arsenic-resistant strains.

To contextualize the effect of the genetic setup characterizing the arsenic-resistant strains, we evaluated the gene expression profile of strains from vineyard-related specimens, using laboratory strains as a reference, grown in laboratory conditions (YPD) and in conditions mimicking must fermentation (synthetic wine must, SWM). With this aim, we carried out a microarray analysis on the RNA extracted from strains resistant and sensitive to arsenic (Table S1). Through this analysis, we were able to identify 269 and 161 genes that were differentially expressed in arsenic-resistant strains compared to arsenic-sensitive strains in YPD and SWM, respectively (Table S9). It should be noted that the *ARR2* and the *ARR3* genes were differentially overexpressed by the arsenic-resistant strains in both YPD and SWM, even if arsenic was not present in the medium (Figure S4). On the other hand, the *ARR1* gene (the transcriptional regulator of the *ARR2* and *ARR3* genes) was differentially expressed in SWM only (Figure S4). The observed overexpression of the *ARR2* and *ARR3* genes in arsenic-resistant strains compared to arsenic-sensitive strains could be ascribed to (i) the higher ‘dosage’ of the genes (multiple copies) or (ii) the new location of the *ARR* cluster, possibly situated in an accessible genomic region available for transcription [42], moving them from a subtelomeric region to a region permitting the ‘constitutive’ expression of the *ARR* genes. If the first hypothesis holds true, there should be a consistent correlation between the expression level of the differentially expressed genes (DEGs) and the depth of coverage calculated from the Illumina reads for those genes. On the contrary, we found only a few positive correlations between copy number and expression (Table S9). In particular, concerning the *ARR* genes, only the expression in SWM and copy number of *ARR2* were positively correlated (Table S9). Hence, the first hypothesis could be confidently excluded. To test the second hypothesis, we searched for correlations among the expression levels of DEGs in the *ARR* cluster and of DEGs located in the regions encompassing the additional copies of the *ARR* cluster (from nt 555,650 to 556,750 in chromosome VIII and from nt 1,083,800 to 1,084,400 in chromosome XV (Figure 2B)) in arsenic-resistant strains. Notably, the expression of *ARR* cluster genes in chromosome VIII was anti-correlated with that of the genes located in proximity to the *ARR* cluster, both in YPD (YHR213W) and in SWM (YHR211W, YHR213W, and YHR218W) (Figure 3B). On the other hand, the expression of genes in the recipient region of chromosome XV was positively correlated with the expression of the *ARR* cluster genes (Figure 3B). In YPD, the genes *FIT2*, *FRM2*, *YAP6*, YHL044W, and *ARR3* were overexpressed in every resistant strain and not differentially expressed or underexpressed (−1 < log2FC < 1) in the vast majority of arsenic-sensitive strains (maximum one sensitive strain) (Figure 3A). In SWM, the gene YHL044W was overexpressed by arsenic-resistant strains only (Figure 3A). In addition, in SWM we observed two genes, *BAP2* and *SUC2*, that were underexpressed only in arsenic-resistant strains (Figure 3A). The genes that were significantly underexpressed in SWM by arsenic-resistant strains were enriched in gene ontologies related to the plasma membrane, while the urea cycle pathway was over-represented in the list of genes underexpressed by the resistant strains in SWM (Table S9). Several GOs and pathways were differentially represented in the list of genes overexpressed in YPD by resistant strains; these were associated with ribosome function and assembly, oxidation–reduction processes, translation, and siderophore transport (Table S9). We investigated whether the genes differentially expressed in strains resistant to arsenic compared to sensitive strains were enriched in arsenic-related genes using the YARG online tool, which investigates a database consisting of genes identified as being associated with arsenic through phenotypic screening and transcriptional profiling [43]. This analysis revealed that the genes overexpressed in YPD by arsenic-resistant strains were enriched in arsenic-associated genes (Table S9). However, among the 109 genes overexpressed in YPD and annotated as being associated with arsenic according to the YARG database, only 31 were reported to be overexpressed in the presence of arsenic in the studies used as a source of data to build the YARG database (Table S9).

## 4. Discussion

We found a few *S. cerevisiae* strains that were isolated from a vineyard environment and resistant to arsenic. These strains were isolated from old vineyards, where the wood used for the espaliers had been treated with arsenic as a preservative. It should be noted that the strains were simultaneously resistant to both forms of tested arsenic (trivalent and pentavalent). The resistance observed in this group of isolates could be also ascribed to the presence of geothermal sources in Tuscany, Italy, an environment rich in heavy metals, including arsenic [44]. In any case, the exposure to arsenic could be one of the factors favoring the selection of microbial-resistant strains. The co-occurrence of arsenite and arsenate resistance in all these strains suggest that it was most probably achieved through changes in the detoxification mechanisms of arsenic rather than as the result of changes in its targets, as observed previously [45]. 

It is already known that the *ARR1*, *ARR2*, and *ARR3* genes are responsible for arsenic resistance, but their presence alone is not sufficient to confer this phenotype; in fact, to our knowledge, most *S. cerevisiae* strains have these genes. Hence, to better understand the molecular mechanisms of resistance, we carried out a combination of high-throughput molecular analyses. First, we used CGH to assess the presence of evident differences at the genomic level between resistant and sensitive strains. As *S. cerevisiae* strains isolated from the same vineyard have been shown to be more genetically similar to each other than to strains isolated from a different location [46], resistant and sensitive strains isolated from the same vineyard were analyzed to reduce the chances of observing differences associated with genetic background rather than with resistance to arsenic. We observed that only 23 genes were consistently over-represented in resistant strains and under-represented in sensitive strains or vice versa. Most of the useful information came from genes that were over-represented in resistant strains, not only because the genes under-represented in resistant strains were all either pseudogenes, dubious ORFs, or genes coding for proteins of unknown functions, but also because the over-represented genes were clearly associated with arsenic. The resistant strains bore additional copies of sixteen genes, among which were the three *ARR* genes. It should be noted that a gene coding a transcription factor involved in conferring resistance to antifungal cycloheximide (*RDS1*) [47] was also over-represented in arsenic-resistant strains. Cycloheximide is produced by *Streptomyces griseus*, an actinomycete commonly found in the soil [48]. This may be a further indication of the role that the environment, shaped by both anthropological and natural activities, can exercise in the selection mechanisms of *S. cerevisiae*. 

The presence of additional copies of the *ARR* genes, identified through CGH analysis, may be the reason why some strains are resistant to arsenic. Whole-genome sequencing confirmed the presence of multiple copies of these genes in the strains resistant to arsenic and also allowed us to predict the location of the additional copies. The combination of NGS, PFGE, and Southern blot hybridization with *ARR1* and *ARR3* probes confirmed that the additional copies of the *ARR* genes were located in chromosomes VIII and XV. Furthermore, transcriptomic analysis allowed us to assess the distinctive traits of the transcriptional profiles of the resistant strains. In particular, we observed that either the presence of multiple copies or the new location was associated with the overexpression of the *ARR* cluster genes in resistant strains, even in the absence of arsenic in the medium. Arr1p has been shown to be regulated by the presence of arsenic at the level of its degradation. Arr1p is always expressed by yeast cells [49,50]; however, in the absence of arsenic, the levels of Arr1p are low, as the protein is degraded through the ubiquitin–proteasome pathway. Contrarily, the presence of arsenic stabilizes Arr1p, either by inhibiting the ubiquitin–proteasome pathway or by stabilizing Arr1p and making it less prone to degradation [51]. Either way, the presence of arsenic is necessary to trigger the effects of Arr1p on the expression of *ARR2* and *ARR3*. It should be noted that the *ARR1* gene was overexpressed only when the resistant strains were grown in SWM, a condition mimicking the wine must fermentation environment. This profile of expression could be ascribed to two different scenarios. These genes could be overexpressed only as a consequence of their higher copy number. In this case, a higher number of copies of the *ARR1* gene would result in a higher amount of Arr1p, with a higher amount of protein potentially ‘surviving’ the ubiquitin–proteasome degradation and being able to trigger the expression of the *ARR2* and *ARR3* genes. However, other genes present in multiple copies in resistant strains were not overexpressed; for instance, the *ARR1* gene was not overexpressed in YPD. In addition, the presence of multiple copies of the *ARR1* gene alone did not imply that the strain was resistant to arsenic, as the strains M28 and M58 showed multiple copies of *ARR1* (observed through CGH) but were sensitive to arsenic. Hence, this scenario may not fully represent the real settings. 

Another characteristic of the *ARR* genes in resistant strains, in addition to being present in higher copy numbers, was the genomic location of their additional copies. In agreement with these observations, a previous study showed a correlation between the resistance to arsenic and the relocation, which was potentially promoted by the subtelomeric presence of a Y’ element, of a copy of the *ARR* cluster in chromosome III [52]. One of the factors determining the level of expression of a gene is the accessibility of the genomic region to transcription factors and/or to the transcription machinery [42]. This could be the case for the additional copies of the *ARR* genes in the subtelomeric region of chromosome XV, where a positive correlation among the expression levels of the *ARR* genes with those of the surrounding genes was found. Thus, the expansion of the cluster could locate the duplicated genes outside the regions where subtelomeric gene silencing occurs. Therefore, the most likely explanation of *ARR* resistance is the relocation of the duplicated *ARR* gene cluster.

## 5. Conclusions

In *S. cerevisiae*, resistance to arsenic is not only a matter of the number of copies of the genes involved in the mechanism of response and resistance, but it is most of all a matter of the location of the genes. In fact, the relocation of the genes responsible for resistance to arsenic outside the subtelomeric gene silencing region has provided some strains with the ability to express the *ARR* cluster even in the absence of arsenic. The unnecessary expression of these genes, despite potentially inducing an energy loss, could ensure the survival of strains that are suddenly exposed to the toxic agent. This is particularly relevant in environments such as old vineyards, where the wood supporting the growth of the vine plants has been treated with CCA (chromated copper arsenate) to prevent rotting [53]. In addition to providing fundamental information to forward our understanding of the genetic mechanisms of arsenic resistance, this study highlights a new and relevant example of the impact of the genomic location of genes on their expression. This work opens new perspectives for the study of the acquisition of resistance (not only to metals, but also to antimicrobial and anti-cancer compounds), which could rely on the same genetic mechanisms highlighted here in the *S. cerevisiae* model and could also be exploited by other organisms.

## Figures and Tables

**Table 1:** Growth of *Saccharomyces cerevisiae* cells (laboratory strains and strains from vineyard-related specimens) in the presence of sodium arsenite and sodium arsenate. Cells (10^3^) of each strain were spotted onto YPD medium supplemented with the reported sodium arsenate or sodium arsenite concentrations. Growth was scored after four days incubation at 30 °C as follows: 0—no growth; +—growth; ++—growth as in control (YPD). * laboratory strains.

Strain	Sodium Arsenite [mM]					Sodium Arsenate [mM]				
	1.25	2.5	5	10	15	6.25	12.5	25	50	75
1014	0	0	0	0	0	0	0	0	0	0
BY4742 *	0	0	0	0	0	0	0	0	0	0
EM93 *	0	0	0	0	0	0	0	0	0	0
M12	0	0	0	0	0	0	0	0	0	0
M28	0	0	0	0	0	0	0	0	0	0
M57	0	0	0	0	0	0	0	0	0	0
SG60	+	+	+	0	0	++	+	0	0	0
SGU89	+	+	+	0	0	++	0	0	0	0
SGU90	+	+	+	0	0	++	+	0	0	0
SGU114	+	+	+	0	0	++	0	0	0	0
SGU406	0	0	0	0	0	0	0	0	0	0
SGU407	0	0	0	0	0	0	0	0	0	0
SK1 *	0	0	0	0	0	0	0	0	0	0
W303 *	0	0	0	0	0	0	0	0	0	0

## Data Availability

The Whole Genome Shotgun data, encompassing the annotated genomes, were deposited in GenBank under the accession numbers WNEH00000000 (1014), WMLL00000000 (SG60), WNEF00000000 (SGU90), and WOFK00000000 (EM93). Raw and normalized expression data were submitted to Gene Expression Omnibus (GEO) under the National Center for Biotechnology Information (NCBI) accession number GSE3021.

## Supplementary Materials

The following supporting information can be downloaded at: https://www.mdpi.com/article/10.3390/ijerph19138119/s1, Supplementary Table S1: Strains used in this study. (In supplementary.pdf) Supplementary Table S2: Growth of *Saccharomyces cerevisiae* strains (laboratory strains or isolated from vineyard-related specimens) in the presence of sodium arsenite and sodium arsenate. (In supplementary.pdf) Supplementary Table S3: list of CGH samples. (In supplementary.pdf) Supplementary Table S4: Sequencing details and stats. (In supplementary.pdf) Supplementary Table S5: Comparison of SNPs among strains resistant and sensitive to arsenic (provided as additional file: TableS5_arsenic-related_SNPs.xls). Supplementary Table S6: Results of circular binary segmentation (CBS) analysis. (Provided as additional file: TableS6_CBS_analysis.xls.) Supplementary Table S7: Identification of genomic regions potentially including the additional copies of the ARR gene cluster (provided as additional file: TableS7_recipient_regions.xls). Supplementary Table S8: Unitigs matching with the ARR gene cluster sequence and details on these partially but uniquely mapped to other genomic locations (provided as additional file: TableS8-unitigs_vs_ARRgenesCluster.xls). Supplementary Table S9: Results of transcriptional analysis on strains resistant or sensitive to arsenic (provided as additional file: TableS9_Expression.xls). Supplementary Figure S1: Schematic representation of the approach used to identify the potential location of the duplicated region. (In supplementary.pdf) Supplementary Figure S2: Potential genomic region recipients of the additional copies of the ARR gene cluster. (In supplementary.pdf) Supplementary Figure S3: Pulsed-field gel electrophoresis (PFGE) and ARR1 or ARR3 hybridization confirm the new location of the ARR gene cluster in strains resistant to arsenic. (In supplementary.pdf) Supplementary Figure S4: Heatmap of genes differentially expressed in YPD or SWM. (In supplementary.pdf) Supplementary results: Details of the assembly unitigs found to match with the ARR gene cluster in SG60 and SGU90. (In supplementary.pdf). References [35,36,37,38,39,40] are cited in the supplementary materials.
