# Supplementary material for: Resistance to Arsenite and Arsenate in Saccharomyces cerevisiae Arises through the Subtelomeric Expansion of a Cluster of Yeast Genes
Source: Int J Environ Res Public Health. 2022 Jul 1;19(13):8119. doi: 10.3390/ijerph19138119 (PMC9266342; doi:10.3390/ijerph19138119)
Supplement: Supplementary file 1 [file ijerph-19-08119-s001.zip › supplementary.pdf]

Supplementary information for

Resistance to Arsenite and Arsenate in *Saccharomyces cerevisiae* Arises through the Subtelomeric Expansion of a Cluster of Yeast Genes

this pdf includes:

Supplementary Tables:

Table S1: Strains used in this study.

Table S2: Growth of *Saccharomyces cerevisiae* cells in the presence of sodium arsenite and sodium arsenate.

Table S3: list of CGH samples.

Table S4: Sequencing details and stats.

Table S5: Comparison of SNPs among strains resistant and sensitive to arsenic [provided as additional file: TableS5_arsenic-related_SNPs.xls].

Table S6: Results of Circular Binary Segmentation (CBS) analysis. [provided as additional file: TableS6_CBS_analysis.xls].

Table S7: Identification of genomic regions potentially including the additional copies of the *ARR* genes cluster [provided as additional file: TableS7_recipient_regions.xls].

Table S8: Unitigs matching with the *ARR* genes cluster sequence and details on these partially but uniquely mapping with other genomic locations [provided as additional file: TableS8-unitigs_vs_ARRgenesCluster.xls].

Table S9: Results of transcriptional analysis on strains resistant or sensitive to arsenic[provided as additional file: TableS9_Expression.xls].

Supplementary Figures:

Figure S1: Schematic representation of the approach used to identify the potential location of the duplicated region.

Figure S2: Genomic regions potential recipients of the additional copies of the *ARR* genes cluster.

Figure S3: Pulsed-Field Gel Electrophoresis (PFGE) and *ARR1* or *ARR3* hybridization confirm the new location of *ARR* genes cluster in strains resistant to arsenic.

Figure S4: Heatmap of genes differentially expressed in YPD or SWM.

Supplementary results:

Details of the assembly unitigs found to match with the *ARR* genes cluster in SG60 and SGU90.

Supplementary Tables

Table S1

Strains used in this study. For each strain, the isolation source and the assays in which the corresponding strain was used are reported.

Stefanini et al. 2012: Stefanini I., Dapporto L., Legras J.L., Calabretta A., Di Paola M., De Filippo C., Viola R., Capretti P., Polsinelli M., Turillazzi S., Cavalieri D. Role of social wasps in Saccharomyces cerevisiae ecology and evolution. Proc Natl Acad Sci U S A. 2012 Aug 14;109(33):13398-403. doi: 10.1073/pnas.1208362109;

Mortimer R.K. and Johnston J.R. (1986) Genealogy of principal strains of the yeast genetic stock center. Genetics 113: 35–43;

Cavalieri et al. 1998: Cavalieri D., Barberio C., Casalone E., Pinzauti F., Sebastiani F., Mortimer R., Poisinelli M. Genetic and Molecular Diversity in *Saccharomyces cerevisiae* natural populations. Food Technology and Biotechnology, 1998, 36(1):45-50;

Kane and Roth 1974: Kane S.M. and Roth R. Carbohydrate metabolism during ascospore development in yeast. J Bacteriol, 1974, 118(1):8-14;

Stern M., Jensen R., Herskowitz I. Five SWI genes are required for expression of the HO gene in yeast. J. Mol. Biol. 1984, 178, 853–868, doi:10.1016/0022-2836(84)90315-2;

Winston F., Dollard C., Ricupero-Hovasse S.L. Construction of a set of convenient Saccharomyces cerevisiae strains that are isogenic to S288C. Yeast, 1995, 11(1):53-5

**Strain name**	**Isolation source**	**Used in assay**	**reference**
1014	Frape for vinsanto fermentation	Phenotypic tests, CGH, whole genome sequencing, SNPs comparison, transcriptomics	Stefanini et al. 2012
EM93	laboratory	Phenotypic tests, CGH, whole genome sequencing, SNPs comparison, transcriptomics	Mortimer and Johnston 1986
M12	Montalcino grape	Phenotypic tests, transcriptomics	Cavalieri et al. 1998
M28	Montalcino grape	Phenotypic tests, SNPs comparison (3 meiotic segregants from the same ascus), transcriptomics	Cavalieri et al. 1998
M57	Montalcino grape	Phenotypic tests, transcriptomics	Cavalieri et al. 1998
SG60	San Giovese must	Phenotypic tests, CGH, whole genome sequencing, SNPs comparison, transcriptomics	Stefanini et al. 2012
SGU89	San Giovese grape	Phenotypic tests, transcriptomics	Stefanini et al. 2012
SGU90	San Giovese grape	Phenotypic tests, CGH, whole genome sequencing, SNPs comparison, transcriptomics	Stefanini et al. 2012
SGU114	San Giovese grape	Phenotypic tests, transcriptomics	Stefanini et al. 2012
SGU406	San Giovese grape	Phenotypic tests, transcriptomics	Stefanini et al. 2012

SGU407	San Giovese grape	Phenotypic tests, transcriptomics	Stefanini et al. 2012
SK1	laboratory	Phenotypic tests, SNPs comparison, transcriptomics	Kane and Roth 1974
W303	laboratory	Phenotypic tests, transcriptomics	Stern et al. 1984
BY4742	laboratory	Phenotypic tests	Winston et al. 1995
BY4743	laboratory	CGH	Winston et al. 1995

Table S2

Growth of *Saccharomyces cerevisiae* cells in the presence of sodium arsenite and sodium arsenate. 100, 1000, and 10000 cells for each tested strain were spotted onto YPD medium supplemented with sodium arsenate or sodium arsenite at the reported concentrations. Growth was scored after 4 days incubation at 30 °C: 0= no growth, += growth, ++= as in control (YPD).

		Sodium arsenite [mM]					Sodium arsenate [mM]				
strain	N.of cells	1.25	2.5	5	10	15	6.25	12.5	25	50	75
1014	10^2	0	0	0	0	0	0	0	0	0	0
	10^3	0	0	0	0	0	0	0	0	0	0
	10^4	0	0	0	0	0	0	0	0	0	0
BY4742	10^2	0	0	0	0	0	0	0	0	0	0
	10^3	0	0	0	0	0	0	0	0	0	0
	10^4	0	0	0	0	0	0	0	0	0	0
EM93	10^2	0	0	0	0	0	0	0	0	0	0
	10^3	0	0	0	0	0	0	0	0	0	0
	10^4	0	0	0	0	0	0	0	0	0	0
M12	10^2	0	0	0	0	0	0	0	0	0	0
	10^3	0	0	0	0	0	0	0	0	0	0
	10^4	0	0	0	0	0	0	0	0	0	0
M28	10^2	0	0	0	0	0	0	0	0	0	0
	10^3	0	0	0	0	0	0	0	0	0	0
	10^4	0	0	0	0	0	0	0	0	0	0
M57	10^2	0	0	0	0	0	0	0	0	0	0
	10^3	0	0	0	0	0	0	0	0	0	0
	10^4	0	0	0	0	0	0	0	0	0	0
SG60	10^2	+	+	+	0	0	++	0	0	0	0
	10^3	+	+	+	0	0	++	+	0	0	0
	10^4	++	++	++	0	0	++	+	+	0	0
SGU89	10^2	+	+	+	0	0	++	0	0	0	0
	10^3	+	+	+	0	0	++	0	0	0	0
	10^4	++	++	++	0	0	++	+	+	0	0
SGU90	10^2	+	+	+	0	0	++	+	0	0	0
	10^3	+	+	+	0	0	++	+	0	0	0
	10^4	++	++	++	0	0	++	+	+	0	0
SGU114	10^2	+	+	+	0	0	++	0	0	0	0
	10^3	+	+	+	0	0	++	0	0	0	0
	10^4	++	++	++	0	0	++	+	+	0	0
SGU406	10^2	0	0	0	0	0	0	0	0	0	0
	10^3	0	0	0	0	0	0	0	0	0	0
	10^4	0	0	0	0	0	0	0	0	0	0
SGU407	10^2	0	0	0	0	0	0	0	0	0	0
	10^3	0	0	0	0	0	0	0	0	0	0
	10^4	0	0	0	0	0	0	0	0	0	0
SK1	10^2	0	0	0	0	0	0	0	0	0	0
	10^3	0	0	0	0	0	0	0	0	0	0
	10^4	0	0	0	0	0	0	0	0	0	0
W303	10^2	0	0	0	0	0	0	0	0	0	0
	10^3	0	0	0	0	0	0	0	0	0	0
	10^4	0	0	0	0	0	0	0	0	0	0

Table S3

List of CGH samples

Accession number	Strain 1 (Cy5)	Strain 2 (Cy3)	replicate
GSM4050898	1014	BY4743	1
GSM4050899	1014	BY4743	2
GSM4050900	EM93	BY4743	1
GSM4050901	EM93	BY4743	2
GSM4050902	M28	BY4743	1
GSM4050903	M28	BY4743	2
GSM4050904	M57	BY4743	1
GSM4050905	M57	BY4743	2
GSM4050906	SG60	BY4743	1
GSM4050907	SG60	BY4743	2
GSM4050908	SGU114	BY4743	1
GSM4050909	SGU114	BY4743	2
GSM4050910	SGU406	BY4743	1
GSM4050911	SGU406	BY4743	2
GSM4050912	SGU407	BY4743	1
GSM4050913	SGU407	BY4743	2
GSM4050914	SGU90	BY4743	1
GSM4050915	SGU90	BY4743	2
GSM4050916	SK1	BY4743	1
GSM4050917	SK1	BY4743	2
GSM4050918	W303	BY4743	1
GSM4050919	W303	BY4743	2

Table S4

Sequencing details and stats. ENA_sample= ID of the data submitted to ArrayExpress; Filtered reads= number of reads remaining after the initial filtering carried out as described in materials and methoods; Coverage (average)= average depth of coverage over the entire genome; Mapped reads= number of reads mapped against the genome of the reference strain S288C; % mapped reads= % of reads mapped against the S288C genome over the total number of reads. (S)= Sensitive to arsenic; (R)= Resistant to arsenic.

strain	Reads_SRA_ID	Filtered reads	Coverage (average)	Mapped reads	Assembly_SRA_ID	% mapped reads
1014 (S)	ERX240908	18,235,568	150	17,545,681	SAMN12640820	96.2
EM93 (S)	ERX240933	18,699,970	153.8	18,005,019	SAMN12640821	96.3
SG60 (R)	ERX240914	7,540,524	90.6	7,318,387	SAMN12640822	97.1
SGU90 (R)	ERX240918	6,889,632	82.7	6,735,090	SAMN12640823	97.8

Note: SRA project ID for sequencing data (also including other strains' sequences)= PRJEB1871; SRA project ID for assembly=PRJNA562447.

**LTP**	**BioSample accession**	
**FZC25**	**SAMN12640820**	1014
**FZC26**	**SAMN12640821**	**EM93**
**FZC27**	**SAMN12640822**	**SG60**
**FZC28**	**SAMN12640823**	**SGU90**

Supplementary Figures

Figure S1: Schematic representation of the approach used to identify the potential location of the duplicated region. Unpaired reads mapping against the region of interests (red arrows) were identified and their read mates (blue arrows) were extracted. Such read mates were then aligned against the whole genome of the reference strain to identify the potential location of the additional copies of the region of interest.

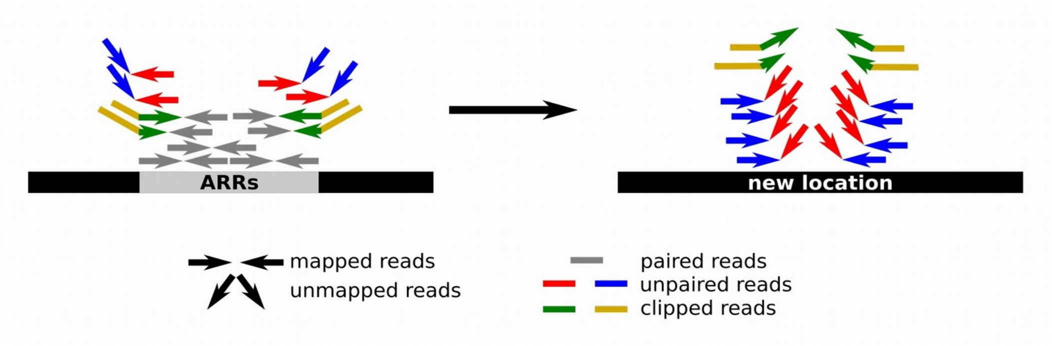

Figure S2:

Genomic regions potential recipients of the additional copies of the *ARR* genes cluster.

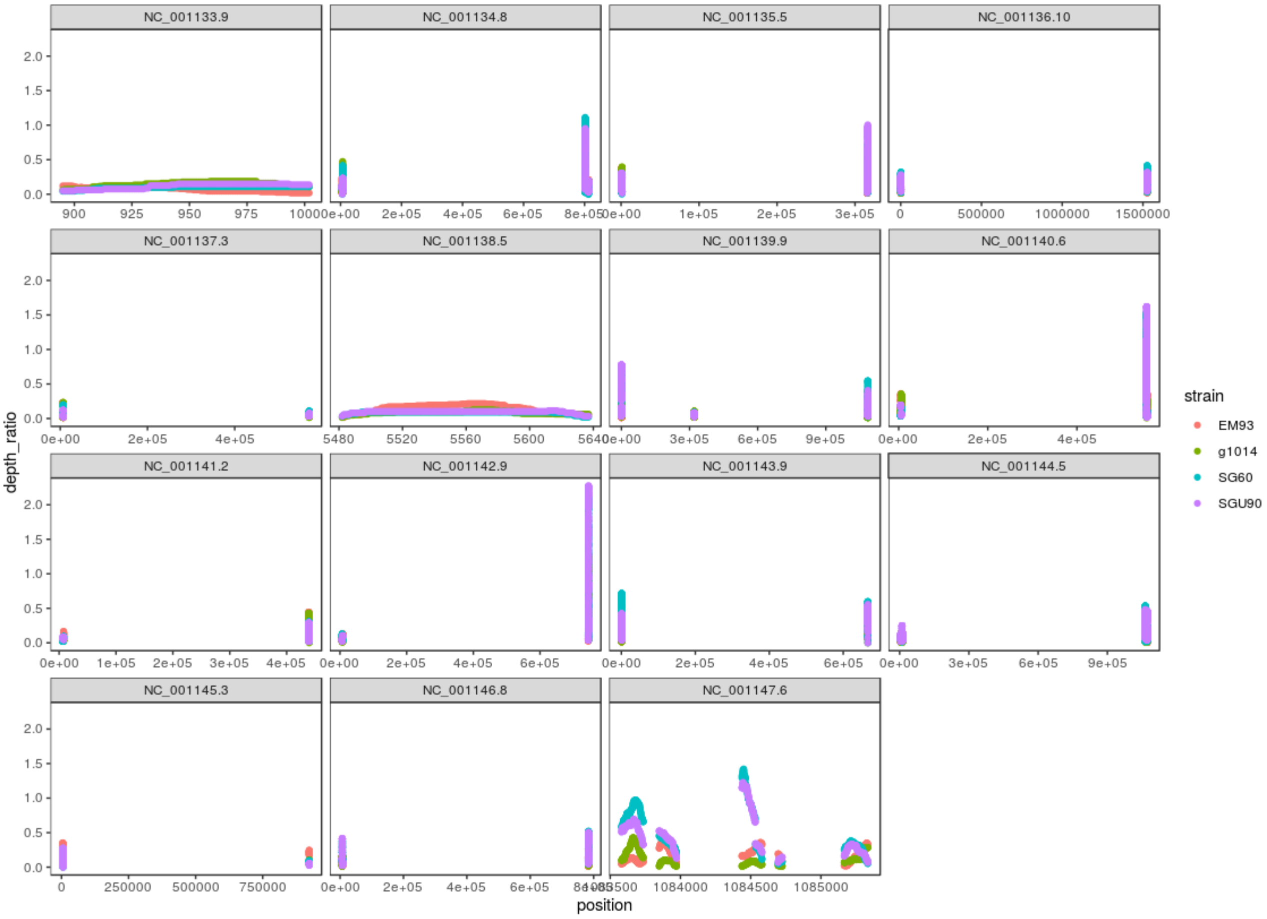

Figure S3:

Pulsed-Field Gel Electrophoresis (PFGE) and ARR1 or ARR3 hybridization confirm the new location of *ARR* genes cluster in strains resistant to arsenic. After the PFGE run, DNA was transferred through southern blot and hybridised with *ARR1* or ARR2 labelled probes. Colored rectangles represent the bands identified through ARR1, ARR3, or both genes hybridization, as indicated in the legend at the bottom of the figure. The tested strain names are listed on top of the figure. Strains resistant to arsenic are SG60, SGU89, SGU90, and SGU114. Missing labels refer to strains not investigated in other assays.

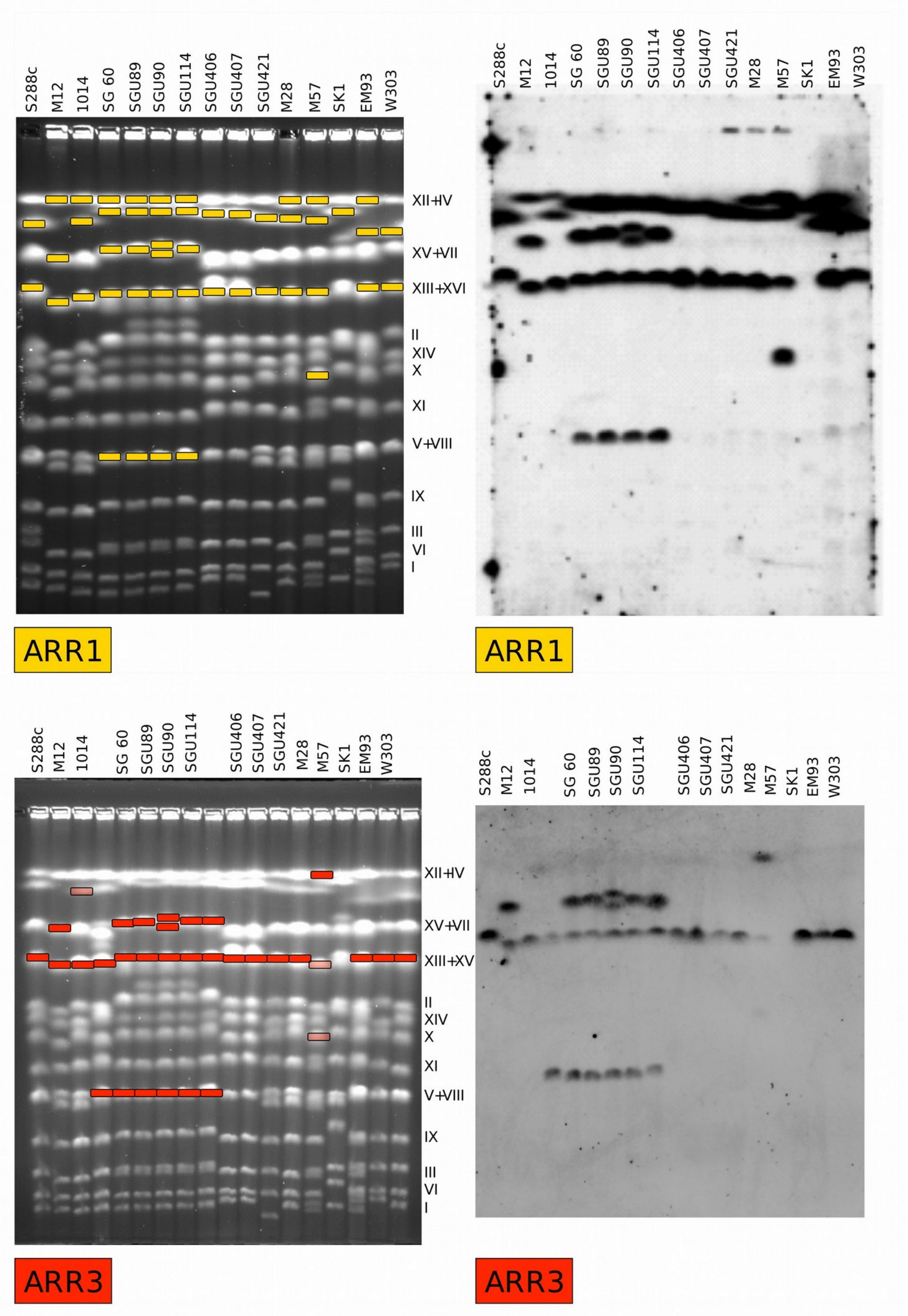

Figure S4:

Heatmap of genes differentially expressed in YPD or SWM. heatmap reporting the expression levels of genes expressed at levels significantly differing among resistant and sensitive strains in either SWM (left) or YPD (rigth). Log2FC was calculated as the log2 transformation of the ratio: expression level (fluorescence of microarray hybridized gene) in the sample/ expression level in the control (BY4743 strains, grown in the same conditions of the sample). Only genes with Log2FC >0.5 or < -0.5 in at least three strains are shown.

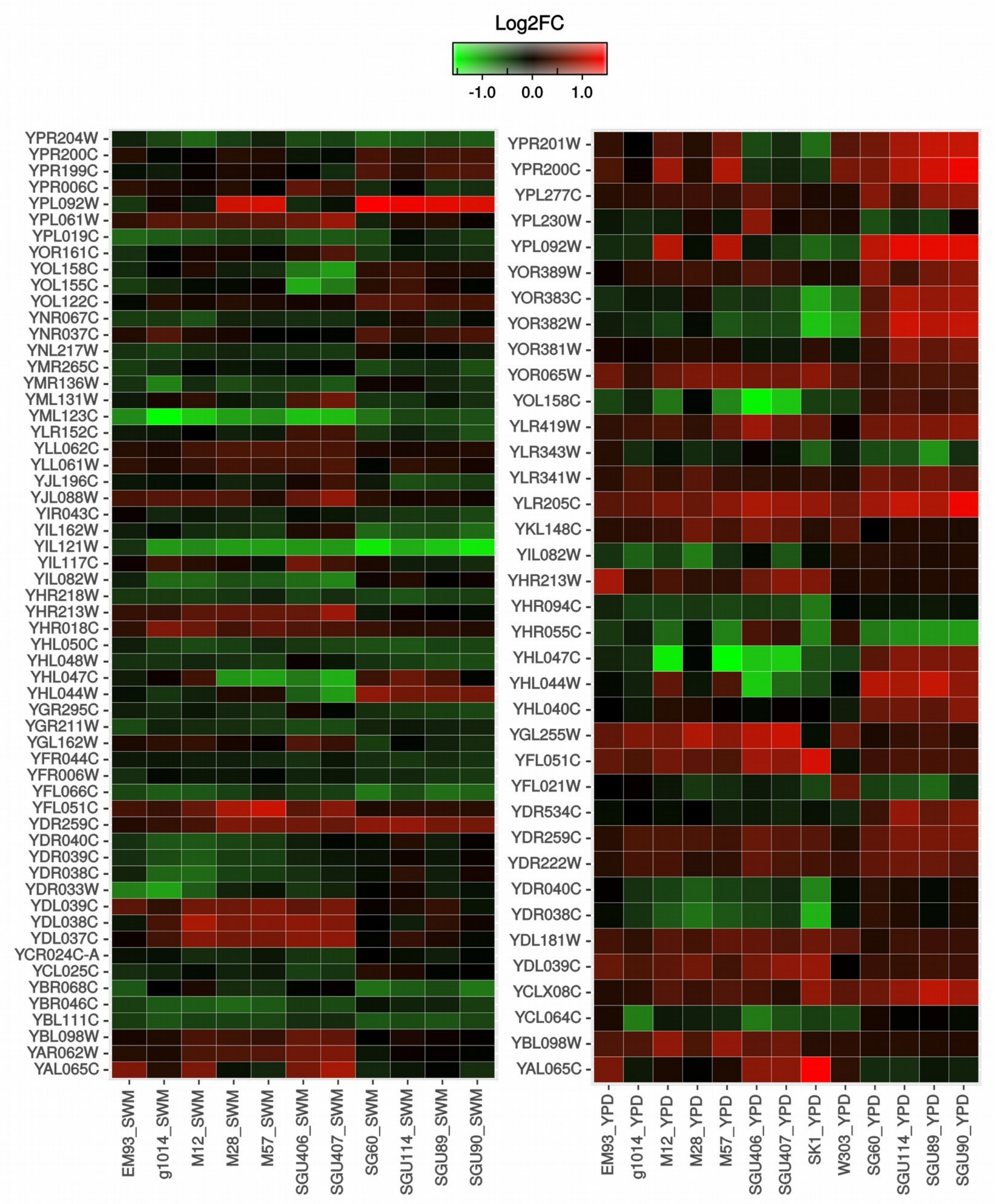

Supplementary results

ARR1

```
SGU90_contig6667      AGATAATGGCAAAACCGCGTGGAAGAAAAGGCGGCAGGAAGCCTTCACTTACTCCACCTA   3273
SG60_contig8180       AGATAATGGCAAAACCGCGTGGAAGAAAAGGCGGCAGGAAGCCTTCACTTACTCCACCTA   3397
EM93_cotig25909       AGATAATGGCAAAACCGCGTGGAAGAAAAGGCGGCAGGAAGCCTTCACTTACTCCACCTA   3750
1014_contig8644       AGATAATGGCAAAACCGCGTGGAAGAAAAGGCGGCAGGAAGCCTTCACTTACTCCACCTA   4080
ARR1                  -----ATGGCAAAACCGCGTGGAAGAAAAGGCGGCAGGAAGCCTTCACTTACTCCACCTA   55
                           *******************************************************

SGU90_contig6667      AAAATAAGAGAGCTGCGCAACTTAGAGCATCCCAAAACGCATTTAGAAAACGAAAGTTGG   3333
SG60_contig8180       AAAATAAGAGAGCTGCGCAACTTAGAGCATCCCAAAACGCATTTAGAAAACGAAAGTTGG   3457
EM93_cotig25909       AAAATAAGAGAGCTGCGCAACTTAGAGCATCCCAAAACGCATTTAGAAAACGAAAGTTGG   3810
1014_contig8644       AAAATAAGAGAGCTGCGCAACTTAGAGCATCCCAAAACGCATTTAGAAAACGAAAGTTGG   4140
ARR1                  AAAATAAGAGAGCTGCGCAACTTAGAGCATCCCAAAACGCATTTAGAAAACGAAAGTTGG   115
                      ************************************************************

SGU90_contig6667      AAAGATTAGAAGAACTAGAGAAGAAAGAAGCTCAGCTAACGGTAACAAATGATCAAATTC   3393
SG60_contig8180       AAAGATTAGAAGAACTAGAGAAGAAAGAAGCTCAGCTAACGGTAACAAATGATCAAATTC   3517
EM93_cotig25909       AAAGATTAGAAGAACTAGAGAAGAAAGAAGCTCAGCTAACGGTAACAAATGATCAAATTC   3870
1014_contig8644       AAAGATTAGAAGAACTAGAGAAGAAAGAAGCTCAGCTAACGGTAACAAATGATCAAATTC   4200
ARR1                  AAAGATTAGAAGAACTAGAGAAGAAAGAAGCTCAGCTAACGGTAACAAATGATCAAATTC   175
                      ************************************************************

SGU90_contig6667      ACATATTAAAGAAGGAAAATGAACTTCTCCATTTTATGTTGAGGAGTCTTTTGACTGAGA   3453
SG60_contig8180       ACATATTAAAGAAGGAAAATGAACTTCTCCATTTTATGTTGAGGAGTCTTTTGACTGAGA   3577
EM93_cotig25909       ACATATTAAAGAAGGAAAATGAACTTCTCCATTTTATGTTGAGGAGTCTTTTGACTGAGA   3930
1014_contig8644       ACATATTAAAGAAGGAAAATGAACTTCTCCATTTTATGTTGAGGAGTCTTTTGACTGAGA   4260
ARR1                  ACATATTAAAGAAGGAAAATGAACTTCTCCATTTTATGTTGAGGAGTCTTTTGACTGAGA   235
                      ************************************************************

SGU90_contig6667      GGAACATGCCTTCTGATGAACGGAACATTAGTAAGGCCTGTTGTGAAGAAAAACCGCCCA   3513
SG60_contig8180       GGAACATGCCTTCTGATGAACGGAACATTAGTAAGGCCTGTTGTGAAGAAAAACCGCCCA   3637
EM93_cotig25909       GGAACATGCCTTCTGATGAACGGAACATTAGTAAGGCCTGTTGTGAAGAAAAACCGCCCA   3990
1014_contig8644       GGAACATGCCTTCTGATGAACGGAACATTAGTAAGGCCTGTTGTGAAGAAAAACCGCCCA   4320
ARR1                  GGAACATGCCTTCTGATGAACGGAACATTAGTAAGGCCTGTTGTGAAGAAAAACCGCCCA   295
                      ************************************************************

SGU90_contig6667      CATGTAACACTCTCGATGGTAGTGTGGTTCTTTCCTCCACATATAATTCCCTTGAAATAC   3573
SG60_contig8180       CATGTAACACTCTCGATGGTAGTGTGGTTCTTTCCTCCACATATAATTCCCTTGAAATAC   3697
EM93_cotig25909       CATGTAACACTCTCGATGGTAGTGTGGTTCTTTCCTCCACATATAATTCCCTTGAAATAC   4050
1014_contig8644       CATGTAACACTCTCGATGGTAGTGTGGTTCTTTCCTCCACATATAATTCCCTTGAAATAC   4380
ARR1                  CATGTAACACTCTCGATGGTAGTGTGGTTCTTTCCTCCACATATAATTCCCTTGAAATAC   355
                      ************************************************************

SGU90_contig6667      AACAATGCTACGTATTTTTCAAGCAACTGCTCTCAGTGTGTGTTGGAAAGAATTGTACAG   3633
SG60_contig8180       AACAATGCTACGTATTTTTCAAGCAACTGCTCTCAGTGTGTGTTGGAAAGAATTGTACAG   3757
EM93_cotig25909       AACAATGCTACGTATTTTTCAAGCAACTGCTCTCAGTGTGTGTTGGAAAGAATTGTACAG   4110
1014_contig8644       AACAATGCTACGTATTTTTCAAGCAACTGCTCTCAGTGTGTGTTGGAAAGAATTGTACAG   4440
ARR1                  AACAATGCTACGTATTTTTCAAGCAACTGCTCTCAGTGTGTGTTGGAAAGAATTGTACAG   415
                      ************************************************************

SGU90_contig6667      TGCCGTCACCGTTGAATTCCTTTGATCGGTCCTTCTATCCCATAGGCTGTACGAATCTCT   3693
SG60_contig8180       TGCCGTCACCGTTGAATTCCTTTGATCGGTCCTTCTATCCCATAGGCTGTACGAATCTCT   3817
EM93_cotig25909       TGCCGTCACCGTTGAATTCCTTTGATCGGTCCTTCTATCCCATAGGCTGTACGAATCTCT   4170
1014_contig8644       TGCCGTCACCGTTGAATTCCTTTGATCGGTCCTTCTATCCCATAGGCTGTACGAATCTCT   4500
ARR1                  TGCCGTCACCGTTGAATTCCTTTGATCGGTCCTTCTATCCCATAGGCTGTACGAATCTCT   475
                      ************************************************************

SGU90_contig6667      CAAATGACATACCAGGCTATTCTTTTCTAAATGATGCAATGAGTGAGATTCATACGTTTG   3753
SG60_contig8180       CAAATGACATACCAGGCTATTCTTTTCTAAATGATGCAATGAGTGAGATTCATACGTTTG   3877
EM93_cotig25909       CAAATGACATACCAGGCTATTCTTTTCTAAATGATGCAATGAGTGAGATTCATACGTTTG   4230
1014_contig8644       CAAATGACATACCAGGCTATTCTTTTCTAAATGATGCAATGAGTGAGATTCATACGTTTG   4560
ARR1                  CAAATGACATACCAGGCTATTCTTTTCTAAATGATGCAATGAGTGAGATTCATACGTTTG   535
                      ************************************************************

SGU90_contig6667      GTGACTTTAATGGGGAACTTGATAGTACCTTTTTGGAGTTTAGTGGGACTGAAATAAAGG   3813
SG60_contig8180       GTGACTTTAATGGGGAACTTGATAGTACCTTTTTGGAGTTTAGTGGGACTGAAATAAAGG   3937
EM93_cotig25909       GTGACTTTAATGGGGAACTTGATAGTACCTTTTTGGAGTTTAGTGGGACTGAAATAAAGG   4290
1014_contig8644       GTGACTTTAATGGGGAACTTGATAGTACCTTTTTGGAGTTTAGTGGGACTGAAATAAAGG   4620
ARR1                  GTGACTTTAATGGGGAACTTGATAGTACCTTTTTGGAGTTTAGTGGGACTGAAATAAAGG   595
                      ************************************************************
```

```
SGU90_contig6667      AGCCAAATAATTTCATTACTGAAAACACTAATGCAATAGAAACAGCGGCAGCGAGCATGG   3873
SG60_contig8180       AGCCAAATAATTTCATTACTGAAAACACTAATGCAATAGAAACAGCGGCAGCGAGCATGG   3997
EM93_cotig25909       AGCCAAATAATTTCATTACTGAAAACACTAATGCAATAGAAACAGCGGCAGCGAGCATGG   4350
1014_contig8644       AGCCAAATAATTTCATTACTGAAAACACTAATGCAATAGAAACAGCGGCAGCGAGCATGG   4680
ARR1                  AGCCAAATAATTTCATTACTGAAAACACTAATGCAATAGAAACAGCGGCAGCGAGCATGG   655
                      ************************************************************

SGU90_contig6667      TGATTAGGCAGGGTTTCCATCCAAGACAGTATTATACAGTGGATGCTTTTGGAGGGGACG   3933
SG60_contig8180       TGATTAGGCAGGGTTTCCATCCAAGACAGTATTATACAGTGGATGCTTTTGGAGGGGACG   4057
EM93_cotig25909       TGATTAGGCAGGGTTTCCATCCAAGACAGTATTATACAGTGGATGCTTTTGGAGGGGACG   4410
1014_contig8644       TGATTAGGCAGGGTTTCCATCCAAGACAGTATTATACAGTGGATGCTTTTGGAGGGGACG   4740
ARR1                  TGATTAGGCAGGGTTTCCATCCAAGACAGTATTATACAGTGGATGCTTTTGGAGGGGACG   715
                      ************************************************************

SGU90_contig6667      TCCTATTAAGTGCGATGGATATTTGGAGTTTTATGAAAGTGCATCCAAAGGTTAACACAT   3993
SG60_contig8180       TCCTATTAAGTGCGATGGATATTTGGAGTTTTATGAAAGTGCATCCAAAGGTTAACACAT   4117
EM93_cotig25909       TCCTATTAAGTGCGATGGATATTTGGAGTTTTATGAAAGTGCATCCAAAGGTTAACACAT   4470
1014_contig8644       TCCTATTAAGTGCGATGGATATTTGGAGTTTTATGAAAGTGCATCCAAAGGTTAACACAT   4800
ARR1                  TCCTATTAAGTGCGATGGATATTTGGAGTTTTATGAAAGTGCATCCAAAGGTTAACACAT   775
                      ************************************************************

SGU90_contig6667      TTGACCTTGAAATTTTGGGAACAGAACTTAAGAAAAGTGCTACGTGTTCTAATTTTGATA   4053
SG60_contig8180       TTGACCTTGAAATTTTGGGAACAGAACTTAAGAAAAGTGCTACGTGTTCTAATTTTGATA   4177
EM93_cotig25909       TTGACCTTGAAATTTTGGGAACAGAACTTAAGAAAAGTGCTACGTGTTCTAATTTTGATA   4530
1014_contig8644       TTGACCTTGAAATTTTGGGAACAGAACTTAAGAAAAGTGCTACGTGTTCTAATTTTGATA   4860
ARR1                  TTGACCTTGAAATTTTGGGAACAGAACTTAAGAAAAGTGCTACGTGTTCTAATTTTGATA   835
                      ************************************************************

SGU90_contig6667      TTTTAATTAGCCTCAAGCATTTCATTAAGGTCTTTTCGTCAAAATTATAATATTCTTCCT   4113
SG60_contig8180       TTTTAATTAGCCTCAAGCATTTCATTAAGGTCTTTTCGTCAAAATTATAATATTCTTCCT   4237
EM93_cotig25909       TTTTAATTAGCCTCAAGCATTTCATTAAGGTCTTTTCGTCAAAATTATAATATTCTTCCT   4590
1014_contig8644       TTTTAATTAGCCTCAAGCATTTCATTAAGGTCTTTTCGTCAAAATTATAATATTCTTCCT   4920
ARR1                  TTTTAATTAGCCTCAAGCATTTCATTAAGGTCTTTTCGTCAAAATTATAA----------   885
                      **************************************************
```

ARR2

```
SG60_contig8180       GAAAACTTGACAATCCAGCAAGCGTAATGGTAAGCTTCATAACGTCTAGGCAACTCAAGG   2739
SGU90_contig6667      GAAAACTTGACAATCCAGCAAGCGTAATGGTAAGCTTCATAACGTCTAGGCAACTCAAGG   2615
EM93_cotig25909       GAAAACTTGACAATCCAGCAAGCGTAATGGTAAGTTTCATAACGTCTAGGCAACTCAAGG   3091
1014_contig8644       GAAAACTTGACAATCCAGCAAGCGTAATGGTAAGTTTCATAACGTCTAGGCAACTCAAGG   3420
ARR2                  --------------------------ATGGTAAGTTTCATAACGTCTAGGCAACTCAAGG   34
                                                ******** *************************

SG60_contig8180       GCCTAATTGAAAATCAGAGGAAGGATTTTCAAGTTGTTGATCTTCGAAGAGAAGATTTTG   2799
SGU90_contig6667      GCCTAATTGAAAATCAGAGGAAGGATTTTCAAGTTGTTGATCTTCGAAGAGAAGATTTTG   2675
EM93_cotig25909       GCCTAATTGAAAATCAGAGGAAGGATTTTCAAGTTGTTGATCTTCGAAGAGAAGATTTTG   3151
1014_contig8644       GCCTAATTGAAAATCAGAGGAAGGATTTTCAAGTTGTTGATCTTCGAAGAGAAGATTTTG   3480
ARR2                  GCCTAATTGAAAATCAGAGGAAGGATTTTCAAGTTGTTGATCTTCGAAGAGAAGATTTTG   94
                      ************************************************************

SG60_contig8180       CACGTGATCATATTACAAACGCTTGGCATGTTCCAGTAACAGCACAGATTACAGAGAAGC   2859
SGU90_contig6667      CACGTGATCATATTACAAACGCTTGGCATGTTCCAGTAACAGCACAGATTACAGAGAAGC   2735
EM93_cotig25909       CACGTGATCATATTACAAACGCTTGGCATGTTCCAGTAACAGCACAGATTACAGAGAAGC   3211
1014_contig8644       CACGTGATCATATTACAAACGCTTGGCATGTTCCAGTAACAGCACAGATTACAGAGAAGC   3540
ARR2                  CACGTGATCATATTACAAACGCTTGGCATGTTCCAGTAACAGCACAGATTACAGAGAAGC   154
                      ************************************************************

SG60_contig8180       AACTGAATCAATTAATTAAAGGTTTATCGGATACTTTCTCAAGTTCTCAATTCGTCAAAG   2919
SGU90_contig6667      AACTGAATCAATTAATTAAAGGTTTATCGGATACTTTCTCAAGTTCTCAATTCGTCAAAG   2795
EM93_cotig25909       AACTGAATCAATTAATTAAAGGTTTATCCGATACTTTCTCAAGTTCTCAATTCGTCAAAG   3271
1014_contig8644       AACTGAATCAATTAATTAAAGGTTTATCCGATACTTTCTCAAGTTCTCAATTCGTCAAAG   3600
ARR2                  AACTGAATCAATTAATTAAAGGTTTATCCGATACTTTCTCAAGTTCTCAATTCGTCAAAG   214
                      **************************** *******************************

SG60_contig8180       TGATATTTCATTGTACTGGGTCCAAGAATAGGGGACCAAAAGTAGCTGCTAAATTCGAAA   2979
SGU90_contig6667      TGATATTTCATTGTACTGGGTCCAAGAATAGGGGACCAAAAGTAGCTGCTAAATTCGAAA   2855
EM93_cotig25909       TGATATTTCATTGTACTGGGTCCAAGAATAGGGGACCAAAAGTAGCTGCTAAATTCGAAA   3331
1014_contig8644       TGATATTTCATTGTACTGGGTCCAAGAATAGGGGACCAAAAGTAGCTGCTAAATTCGAAA   3660
ARR2                  TGATATTTCATTGTACTGGGTCCAAGAATAGGGGACCAAAAGTAGCTGCTAAATTCGAAA   274
                      ************************************************************

SG60_contig8180       CCTACTTACAAGAAGAAGATATTACAAGTAAGTTTGAGAGTTGCATCCTCGTTGGAGGTT   3039
SGU90_contig6667      CCTACTTACAAGAAGAAGATATTACAAGTAAGTTTGAGAGTTGCATCCTCGTTGGAGGTT   2915
```

```
EM93_cotig25909      CCTACTTACAAGAAGAAGATATTACAAGTAAGTTTGAGAGTTGCATCCTCGTTGGAGGTT   3391
1014_contig8644      CCTACTTACAAGAAGAAGATATTACAAGTAAGTTTGAGAGTTGCATCCTCGTTGGAGGTT   3720
ARR2                 CCTACTTACAAGAAGAAGATATTACAAGTAAGTTTGAGAGTTGCATCCTCGTTGGAGGTT   334
                     ************************************************************

SG60_contig8180      TTTACGCTTGGGAGACCCATTGTAGAGAGAGTAACCTTAAATTGATTGTTAGTGGTTGAG   3099
SGU90_contig6667     TTTACGCTTGGGAGACCCATTGTAGAGAGAGTAACCTTAAATTGATTGTTAGTGGTTGAG   2975
EM93_cotig25909      TTTACGCTTGGGAGACCCATTGTAGAGAGAGTAACCTTAAATTGATTGTTAGTGGTTGAG   3451
1014_contig8644      TTTACGCTTGGGAGACCCATTGTAGAGAGAGTAACCTTAAATTGATTGTTAGTGGTTGAG   3780
ARR2                 TTTACGCTTGGGAGACCCATTGTAGAGAGAGTAACCTTAAATTGATTGTTAGTGGTTGA-   393
                     ***********************************************************
```

ARR3

```
SGU90_contig6667     ATTAATTTCTATTGTTCCATATATAATATGGTTTAAGGATCCTCGCGACTATTGCCAAAA   1175
SG60_contig8180      ATTAATTTCTATTGTTCCATATATAATATGGTTTAAGGATCCTCGCGACTATTGCCAAAA   1299
EM93_cotig25909      ATTAATTTCTATTGTTCCATATATAATATGGTTTAAGGATCCTCGCGACTATTGCCAAAA   1651
1014_contig8644      ATTAATTTCTATTGTTCCATATATAATATGGTTTAAGGATCCTCGCGACTATTGCCAAAA   1980
ARR3_YPR201W         -TTAATTTCTATTGTTCCATATATAATATGGTTTAAGGATCCTCGCGACTATTGCCAAAA   59
                      ***********************************************************

SGU90_contig6667     TCAATAAAATTGGAACTTCTAGCAACGGCCCAAATGTTGCAGCTATTGCTTGCTTGCTAT   1235
SG60_contig8180      TCAATAAAATTGGAACTTCTAGCAACGGCCCAAATGTTGCAGCTATTGCTTGCTTGCTAT   1359
EM93_cotig25909      TCAATAAAATTGGAACTTCTAGCAACGGCCCAAATGTTGCAGCTATTGCTTGCTTGCTAT   1711
1014_contig8644      TCAATAAAATTGGAACTTCTAGCAACGGCCCAAATGTTGCAGCTATTGCTTGCTTGCTAT   2040
ARR3_YPR201W         TCAATAAAATTGGAACTTCTAGCAACGGCCCAAATGTTGCAGCTATTGCTTGCTTGCTAT   119
                     ************************************************************

SGU90_contig6667     TGTTACCATATAAGGAAATAGCAATTGCCAGGGATAGTTCAAAATTATTTGAAGCCATAG   1295
SG60_contig8180      TGTTACCATATAAGGAAATAGCAATTGCCAGGGATAGTTCAAAATTATTTGAAGCCATAG   1419
EM93_cotig25909      TGTTACCATATAAGGAAATAGCAATTGCCAGGGATAGTTCAAAATTATTTGAAGCCATAG   1771
1014_contig8644      TGTTACCATATAAGGAAATAGCAATTGCCAGGGATAGTTCAAAATTATTTGAAGCCATAG   2100
ARR3_YPR201W         TGTTACCATATAAGGAAATAGCAATTGCCAGGGATAGTTCAAAATTATTTGAAGCCATAG   179
                     ************************************************************

SGU90_contig6667     TGAAACATTGCGTCATCGTAATAGAAAAGCTAGCTTCACAAGACTTTCTTCCCCAGACCC   1355
SG60_contig8180      TGAAACATTGCGTCATCGTAATAGAAAAGCTAGCTTCACAAGACTTTCTTCCCCAGACCC   1479
EM93_cotig25909      TGAAACATTGCGTCATCGTAATAGAAAAGCTAGCTTCACAAGACTTTCTTCCCCAGACCC   1831
1014_contig8644      TGAAACATTGCGTCATCGTAATAGAAAAGCTAGCTTCACAAGACTTTCTTCCCCAGACCC   2160
ARR3_YPR201W         TGAAACATTGCGTCATCGTAATAGAAAAGCTAGCTTCACAAGACTTTCTTCCCCAGACCC   239
                     ************************************************************

SGU90_contig6667     TCTTTAAAAGTAGTTCTTGGTCACAGCTACATTCTCTTTGTGTATCACTCCTAGATATTG   1415
SG60_contig8180      TCTTTAAAAGTAGTTCTTGGTCACAGCTACATTCTCTTTGTGTATCACTCCTAGATATTG   1539
EM93_cotig25909      TCTTTAAAAGTAGTTCTTGGTCACAGCTACATTCTCTTTGTGTATCACTCCTAGATATTG   1891
1014_contig8644      TCTTTAAAAGTAGTTCTTGGTCACAGCTACATTCTCTTTGTGTATCACTCCTAGATATTG   2220
ARR3_YPR201W         TCTTTAAAAGTAGTTCTTGGTCACAGCTACATTCTCTTTGTGTATCACTCCTAGATATTG   299
                     ************************************************************

SGU90_contig6667     ATAAGTACCTCATTAATGCGAAGGTCAAAAACCATGCAATAAAGAAGTAAAGCACCAATG   1475
SG60_contig8180      ATAAGTACCTCATTAATGCGAAGGTCAAAAACCATGCAATAAAGAAGTAAAGCACCAATG   1599
EM93_cotig25909      ATAAGTACCTCATTAATGCGAAGGTCAAAAACCATGCAATAAAGAAGTAAAGCACCAATG   1951
1014_contig8644      ATAAGTACCTCATTAATGCGAAGGTCAAAAACCATGCAATAAAGAAGTAAAGCACCAATG   2280
ARR3_YPR201W         ATAAGTACCTCATTAATGCGAAGGTCAAAAACCATGCAATAAAGAAGTAAAGCACCAATG   359
                     ************************************************************

SGU90_contig6667     GGACAAAGCACAATATTGCAGAACCAATTTCGTGGATAAATTGATAACCTCTACTAATAA   1535
SG60_contig8180      GGACAAAGCACAATATTGCAGAACCAATTTCGTGGATAAATTGATAACCTCTACTAATAA   1659
EM93_cotig25909      GGACAAAGCACAATATTGCAGAACCAATTTCGTGGATAAATTGATAACCTCTACTAATAA   2011
1014_contig8644      GGACAAAGCACAATATTGCAGAACCAATTTCGTGGATAAATTGATAACCTCTACTAATAA   2340
ARR3_YPR201W         GGACAAAGCACAATATTGCAGAACCAATTTCGTGGATAAATTGATAACCTCTACTAATAA   419
                     ************************************************************

SGU90_contig6667     AAATAACAAATAAAGTGTAATGAAATCCGATCATTGCCCATGGAGAAATAAATCTCAAAA   1595
SG60_contig8180      AAATAACAAATAAAGTGTAATGAAATCCGATCATTGCCCATGGAGAAATAAATCTCAAAA   1719
EM93_cotig25909      AAATAACAAATAAAGTGTAATGAAATCCGATCATTGCCCATGGAGAAATAAATCTCAAAA   2071
1014_contig8644      AAATAACAAATAAAGTGTAATGAAATCCGATCATTGCCCATGGAGAAATAAATCTCAAAA   2400
ARR3_YPR201W         AAATAACAAATAAAGTGTAATGAAATCCGATCATTGCCCATGGAGAAATAAATCTCAAAA   479
                     ************************************************************

SGU90_contig6667     TGTATTTTTCATAATTACTTTTACCAGCTATGGTAAGACTTCCCAAACGTATGATAATGC   1655
SG60_contig8180      TGTATTTTTCATAATTACTTTTACCAGCTATGGTAAGACTTCCCAAACGTATGATAATGC   1779
EM93_cotig25909      TGTATTTTTCATAATTACTTTTACCAGCTATGGTAAGACTTCCCAAACGTATGATAATGC   2131
1014_contig8644      TGTATTTTTCATAATTACTTTTACCAGCTATGGTAAGACTTCCCAAACGTATGATAATGC   2460
```

```
ARR3_YPR201W          TGTATTTTTCATAATTACTTTTACCAGCTATGGTAAGACTTCCCAAACGTATGATAATGC   539
                      ************************************************************

SGU90_contig6667      CAATTCCCAGTGGTATGCCGAGAAAAACTCCGACAGACTTTGCAACCTCTTCGAATAATA   1715
SG60_contig8180       CAATTCCCAGTGGTATGCCGAGAAAAACTCCGACAGACTTTGCAACCTCTTCGAATAATA   1839
EM93_cotig25909       CAATTCCCAGTGGTATGCCGAGAAAAACTCCGACAGACTTTGCAACCTCTTCGAATAATA   2191
1014_contig8644       CAATTCCCAGTGGTATGCCGAGAAAAACTCCGACAGACTTTGCAACCTCTTCGAATAATA   2520
ARR3_YPR201W          CAATTCCCAGTGGTATGCCGAGAAAAACTCCGACAGACTTTGCAACCTCTTCGAATAATA   599
                      ************************************************************

SGU90_contig6667      CCCTATTTGAAGTATTCAGGTGGTCATGAGAAATAACATAACAGTAAAATATCTGCAATG   1775
SG60_contig8180       CCCTATTTGAAGTATTCAGGTGGTCATGAGAAATAACATAACAGTAAAATATCTGCAATG   1899
EM93_cotig25909       CCCTATTTGAAGTATTCAGGTGGTCATGAGAAATAACATAACAGTAAAATATCTGCAATG   2251
1014_contig8644       CCCTATTTGAAGTATTCAGGTGGTCATGAGAAATAACATAACAGTAAAATATCTGCAATG   2580
ARR3_YPR201W          CCCTATTTGAAGTATTCAGGTGGTCATGAGAAATAACATAACAGTAAAATATCTGCAATG   659
                      ************************************************************

SGU90_contig6667      GTGCATATAATACCATCTGTAAAAGCGAGTTTGTAATAACAAGCACGACGCAGAGATCAT   1835
SG60_contig8180       GTGCATATAATACCATCTGTAAAAGCGAGTTTGTAATAACAAGCACGACGCAGAGATCAT   1959
EM93_cotig25909       GTGCATATAATACCATCTGTAAAAGCGAGTTTGTAATAACAAGCACGACGCAGAGATCAT   2311
1014_contig8644       GTGCATATAATACCATCTGTAAAAGCGAGTTTGTAATAACAAGCACGACGCAGAGATCAT   2640
ARR3_YPR201W          GTGCATATAATACCATCTGTAAAAGCGAGTTTGTAATAACAAGCACGACGCAGAGATCAT   719
                      ************************************************************

SGU90_contig6667      TGTCTCCTCCAGCAATCTGATTCCAAATTAGCACCATGGCAATGCATCTAGCTACTCCGA   1895
SG60_contig8180       TGTCTCCTCCAGCAATCTGATTCCAAATTAGCACCATGGCAATGCATCTAGCTACTCCGA   2019
EM93_cotig25909       TGTCTCCTCCAGCAATCTGATTCCAAATTAGCACCATGGCAATGCATCTAGCTACTCCGA   2371
1014_contig8644       TGTCTCCTCCAGCAATCTGATTCCAAATTAGCACCATGGCAATGCATCTAGCTACTCCGA   2700
ARR3_YPR201W          TGTCTCCTCCAGCAATCTGATTCCAAATTAGCACCATGGCAATGCATCTAGCTACTCCGA   779
                      ************************************************************

SGU90_contig6667      TCATAATAATGCCTTGACGGTATTCCTTATAATCGAATAGCGCCATCCACGCCAATGCTG   1955
SG60_contig8180       TCATAATAATGCCTTGACGGTATTCCTTATAATCGAATAGCGCCATCCACGCCAATGCTG   2079
EM93_cotig25909       TCATAATAATGCCTTGACGGTATTCCTTATAATCGAATAGCGCCATCCACGCCAATGCTG   2431
1014_contig8644       TCATAATAATGCCTTGACGGTATTCCTTATAATCGAATAGCGCCATCCACGCCAATGCTG   2760
ARR3_YPR201W          TCATAATAATGCCTTGACGGTATTCCTTATAATCGAATAGCGCCATCCACGCCAATGCTG   839
                      ************************************************************

SGU90_contig6667      TCATCAACAAAGGACCGATGACCCAATTCAAAAATAACGAGAGGGCTAGTTGCTTCCTTA   2015
SG60_contig8180       TCATCAACAAAGGACCGATGACCCAATTCAAAAATAACGAGAGGGCTAGTTGCTTCCTTA   2139
EM93_cotig25909       TCATCAACAAAGGACCGATGACCCAATTCAAAAATAACGAGAGGGCTAGTTGCTTCCTTA   2491
1014_contig8644       TCATCAACAAAGGACCGATGACCCAATTCAAAAATAACGAGAGGGCTAGTTGCTTCCTTA   2820
ARR3_YPR201W          TCATCAACAAAGGACCGATGACCCAATTCAAAAATAACGAGAGGGCTAGTTGCTTCCTTA   899
                      ************************************************************

SGU90_contig6667      TATAGCTCCTGTAGAAGTACTTGTGAATAGACTCCCAGGAAACTTTGCAGATCGGGGGAA   2075
SG60_contig8180       TATAGCTCCTGTAGAAGTACTTGTGAATAGACTCCCAGGAAACTTTGCAGATCGGGGGAA   2199
EM93_cotig25909       TATAGCTCCTGTAGAAGTACTTGTGAATAGACTCCCAGGAAACTTTGCAGATCGGGGGAA   2551
1014_contig8644       TATAGCTCCTGTAGAAGTACTTGTGAATAGACTCCCAGGAAACTTTGCAGATCGGGGGAA   2880
ARR3_YPR201W          TATAGCTCCTGTAGAAGTACTTGTGAATAGACTCCCAGGAAACTTTGCAGATCGGGGGAA   959
                      ************************************************************

SGU90_contig6667      TCATCATTACAATCATACCAACAGTCAAAGGAATGGACACTCCCATTAGATTGGGATGAC   2135
SG60_contig8180       TCATCATTACAATCATACCAACAGTCAAAGGAATGGACACTCCCATTAGATTGGGATGAC   2259
EM93_cotig25909       TCATCATTACAATCATACCAACAGTCAAAGGAATGGACACTCCCATTAGATTGGGATGAC   2611
1014_contig8644       TCATCATTACAATCATACCAACAGTCAAAGGAATGGACACTCCCATTAGATTGGGATGAC   2940
ARR3_YPR201W          TCATCATTACAATCATACCAACAGTCAAAGGAATGGACACTCCCATTAGATTGGGATGAC   1019
                      ************************************************************

SGU90_contig6667      CTTCAGCGTCAAAAGTGTGACGGGAAGAAGGCACATAGACAGAAATTATTACTGCAATGA   2195
SG60_contig8180       CTTCAGCGTCAAAAGTGTGACGGGAAGAAGGCACATAGACAGAAATTATTACTGCAATGA   2319
EM93_cotig25909       CTTCAGCGTCAAAAGTGTGACGGGAAGAAGGCACATAGACAGAAATTATTACTGCAATGA   2671
1014_contig8644       CTTCAGCGTCAAAAGTGTGACGGGAAGAAGGCACATAGACAGAAATTATTACTGCAATGA   3000
ARR3_YPR201W          CTTCAGCGTCAAAAGTGTGACGGGAAGAAGGCACATAGACAGAAATTATTACTGCAATGA   1079
                      ************************************************************

SGU90_contig6667      TTATGGAGAGAATTATAGTAAATGGCAACATCAAGTCAAGCCATGACAATGACTTGATCG   2255
SG60_contig8180       TTATGGAGAGAATTATAGTAAATGGCAACATCAAGTCAAGCCATGACAATGACTTGATCG   2379
EM93_cotig25909       TTATGGAGAGAATTATAGTAAATGGCAACATCAAGTCAAGCCATGACAATGACTTGATCG   2731
1014_contig8644       TTATGGAGAGAATTATAGTAAATGGCAACATCAAGTCAAGCCATGACAATGACTTGATCG   3060
ARR3_YPR201W          TTATGGAGAGAATTATAGTAAATGGCAACATCAAGTCAAGCCATGACAATGACTTGATCG   1139
                      ************************************************************

SGU90_contig6667      TAGTCAGTATATCGGTGCGATTCACCATATTAACCTTAGAAGGTACCGAATTTTCACTTT   2315
SG60_contig8180       TAGTCAGTATATCGGTGCGATTCACCATATTAACCTTAGAAGGTACCGAATTTTCACTTT   2439
```

```
EM93_cotig25909       TAGTCAGTATATCGGTGCGATTCACCATATTAACCTTAGAAGGTACCGAATTTTCACTTT   2791
1014_contig8644       TAGTCAGTATATCGGTGCGATTCACCATATTAACCTTAGAAGGTACCGAATTTTCACTTT   3120
ARR3_YPR201W          TAGTCAGTATATCGGTGCGATTCACCATATTAACCTTAGAAGGTACCGAATTTTCACTTT   1199
                      ************************************************************

SGU90_contig6667      TTTGATCTTCTGACATTATTCTACTAACCTGATGATTTGTTGGTTGGGTTCTCTTGAATT   2375
SG60_contig8180       TTTGATCTTCTGACATTATTCTACTAACCTGATGATTTGTTGGTTGGGTTCTCTTGAATT   2499
EM93_cotig25909       TTTGATCTTCTGACATTATTCTACTAACCTGATGATTTGTTGGTTGGGTTCTCTTGAATT   2851
1014_contig8644       TTTGATCTTCTGACATTATTCTACTAACCTGATGATTTGTTGGTTGGGTTCTCTTGAATT   3180
ARR3_YPR201W          TTTGATCTTCTGACAT--------------------------------------------   1215
                      ****************
```

Details of the assembly unitigs found to match with the *ARR* genes cluster in SG60 and SGU90. 1014 and EM93 (sensitive strains) unitigs are not reported because if partially matching with chr XVI the portion of the unitig not matching with chr XVI matched with multiple chromosomes. After the sequence of each relevant unitig (formatted as described in the color legend), the coordinates of the mapping are reported, with the position of the *ARR* genes cluster sequence being referred to the *ARR* genes cluster (considered to stat from nucleotide 925,709 of chromosome XVI).

Color legend:
Mapping vs *ARR* genes cluster
Mapping vs chr VIII
Mapping vs chr XV

>SG60_unitig 1787
TACAGTATTACTGATGATATGTTATTTAGTGCTGTCCGTATGTCCTCCTGTTCTTTTTTGGGCTACAATTTAATTGATGGGGTGTTATG
CTTACGATATTTCAATTATGTTAATATTTAGTATGTTTATATCTTCTCATTAATGAATGGTTAATTTTTA
[matches with chr VIII from 555,989 to 556,053]
[matches with *ARR* genes cluster from 16,636 to 16,730]

>SG60_unitig5436
TTAGATTTGAATTCAGAACCGCTTCTGCTCAACTAGAAGGTGGCGTTCATAATTTACATTCTTACGAAAAGCGTTTACATAACTGAATG
ATAAAATGGCATCATCAATACAATATTACTGATGATATGTTATTTAGTGCTGTACGTATGCCCTCCTGTTCTTTTTTGGGCTACAATTT
AATTGATGGAGTGTTATGTTTACGATATTTCAATTATGTTAATATTTAGTATGTTTATATCTTCTCATTAATGAATAGTTAATTTTTAC
GTTTAGGTGA
[mapping vs chr VIII (ref) from 555882 to 556053]
[matches with *ARR* genes cluster from 16,636 to 16,741]

>SG60_unitig6295
TCACCTAAACGTAAAAATTAACTATTCATTAATGAGAAGATATAAACATACTAAATATTAACATAATTGAAATATCGTAAACATAACAC
CCCATCAATTAAATTGTAGCCCAAAAAAGAACAGGAGGACATACGGACAGCACTAAATAACATATCATCAGTAATACTGTA
[mapping vs chr VIII (ref) from 556052 to 555989]
[matches with *ARR* genes cluster from 16,636 to 16,741]

>SG60_unitig4277
TTTTTGAGCTTTCTAATAGTGCTCCTGAGCAAAAGATCCGGCATCTACGCTCAATCTCTATGTATCACTTTATCAAGAAATATGTATCT
GATATCAAGAATAGAAATGTAAGAAAATGAAATTAGATTATACAAGTTGTATTGAGAAAAGTGAAGAGAAACCTGTGAAATATGTTAAA
ATCACAACACAACATTCTTGGCAAGTTTGTTCTTGTATTGTAAAATACTACCATCCCATCTTGTAGCAGTTTTGTGTTCAACAACAAAA
ATATCCTTTGCAATTTTATCCAGCAATCTGAAATCGTGAGATACGACAACAACACCACCGTTAAATTCAAGGATAGCTTCAGCCAAAGA
ATCAATAGTTGGAATGTCCAGACCGTTAGTAGGTTCATCCAGTAGTAGAACATTGGGCTGCTCAAGGGCTAGCAATGCAAAAACAACAC
GCGAACGTTGTCCTTCAGATAATGTACCCATTTGGACCGTTTGACCTTCACCTGTTAAACCGTAACGTCCTAATTGACCCCTCCAGTAT
TGGAAATCTTGTGAGATGTTTGGATATTTGTCACGAACAAATTCCAGGGCAGATTTTGTTAAATCTAATTGATCTTGAGAATGTTGTGA
GTAAACTCCCAATTTAACATGTGTACGCCCTAAGAAACAGTGATATTCTATAGCCTGATTTTGCCCTCGTTGGTTTTCTTAGGGCATAT
TGCCTTCATGTATATTGAAGATTTTCTCTATGCTTTGTGTCTTAAAGCATAATTAAACTCAACCAACCAACGATGTTTCAACAGCCAGA
GGTTTCTTTTCACTTTTGAAAGGTTAAAGCAATACTCCATTGAGGACCAGTTCATTCTCACAAAGGCTTTGTAGACATATGTCTTGAAT
ACGTAGTGAGCAACTTGTGGAATGCTTTCTTCTCACCGGTTATGGGTAAATCCATTTAATCGCTTCTCTTTAATTTACCACGTACGGAT
GTAGCCTCTAGAAGATCTCACTCTGATTCAATGCTCTAGTACCTCTAGTAATTATGAATTGAAAATCATGACTTAGGAAGGATTGACTG
CGGACGTCGAAATCTGTGTGTTCCAATACCTCGGAGTTGCCTATAAAGGACCAGTAAAGGTTGTTTTTAAAAGAATCCAGTTAGAGTTC
AGCCCTTTCAGATCTTTGGGTAAGTACGGAAATTTTGAAATCCGCAACTTGAAAGTGTGCAATAGATACGTTGTTCATTTAGAAGTGGG
CATACAAACAATCTTTCCTTTCGGATGATACTGCTTGGACATGGTACGCTTTAGAGTTTTTATTCAGCTTCATGAAAAAAAATGCCATA
CTGTCGCTAAATATTGAAGACGTCGGTAAAGGGCCTGCCATGTGCTCACATTTAATCCTACTCCTTATAATTATGGCAGTGATTGATCG
GGGTGGCACTAAATACCATATTAGGAACAATACATTCCCTTACGTAACGGAAACCCTAAAACGTCTTTTTTTGCAGGCTTAAATAATCA
CAGTAGTTGCAGACGGCCTTATAGGCATATTGCGGGCTTTGCTATTCTGAAGGATGAGAAGTGAAAAAAATTGCACTTTTATGAATCAA
TAATTACTACAAAGAACTTCCTGTTGACAAATCGAGGGAAGCATCCCACCTTTCAAAGTTCATAGATAAGTTACGAAAGGTCTCTGGTG
AAAAATGTTCAAATCCTGCACCAGAGCTTTCTAATTCAATTAAGGATGGCAAATTCGCATTAAGTTCGTCCTGACGCAGTTCTTCAGAA
AGTGGAGCATTATCTTGAAGAGTTGAAATTTTTTTTGCATGCAGTGATTCATTTTCTACTAGGGAATTTTCACCGTCTTCAGTAATTGT
GGTGCGGTTAGATTGTAAAGATTTCATCGAAGATTCTTCCCTGTTCTGTCTCTCCTTTTCTGCCTCGAACAGAATACTATCGAGTTCCT
TCAGCCGACTGTAATTATCCAATGCCGTAATATTGCCGGCTTTACACATTGAGGCATTCAAGGACAACGCTATCTGTAGCTCAGGAAAC
GCGTCCCCATTCAATGTTTCCATAACCAGCATCAAAATGGATGAAAAGCAGCATTGCGCTTCGAGGAACCCAAAAATGCAAAACCAACC
AGAATTCCAAAGGTTGTTCAAAATACAGATATTTGTTACAGCAGCGAGAACACATTTGACTTTGGCATCCTGAAGTTCATCAGTAACTC
TTATAGTAGATTTCAACAGTGAAAGGTAAAGCGGCCGACCAGTCACTATGATTATTTGATGGTAACGCAAAGTTAAAACCGCTATGGAC
CTATTTGACTCAATAATATCAGGCAAAACCCTTAATTCTGGGTCAAGACTATCTCGAAAAAGTTCTAACTGCTTTAAACTGTCGACTAT
GTTTGTTATGAAATCATTGTCCCCAGAAGTGACATAAACTTTCTTCATTACCGAGCCAATGTGAAGAGCTAATTTCGTGTTCGCAATTA
AAATTTTTGGATCAAACCCGTCCTCGTTAGATATATTTTTATCTGGTACATCAAGGTCCATATAATCCACGTTAAAATGAGGCGGCAAG
CCGAGTCTAGTAGCCCATAGGATGTCCATAAAAAATGCAGTCCACCATACTTTCTTTCTTGCATCTTGTTCTGCCTTCGGTCGTCCAAC
ATTGCTTACTTCTCTATGCATCCCTAACATTATAGCAGTCGATAAGGCGAGCCCAGTGTAGGTGAAACCAGAATTTACTCTATTCCATC

CTTGAGAGTATATAGCAAACAAAAGCAATGTTCTGACTGTATCTAAGGTGGGCTCTTCAATCGGTTCATGGAATAATGTGAGGGCTGTC
GAAAAAAAGCTTCTTCCAGGTACTTTTTTGATACCAACGCCTGCAGCATCGTTTGTATACTGCTCCCCTACTGCAAAGGTTACCATGAG
GTATGCAAGCCAGCTAGGATCTTGCGATTGGACATCTCTATAGGTTTCTTCAAGTCGGACATGGAACTCAGTGATTGAGAATAAACCAT
ACTCTTTTGCAAAGAACTGGTAGACTCTGTGGATCAGTTCTGATGCATATGCTCTTTCAGGTAAGATAGCTTTCTGATTATCACCTCTT
TCTTGATCATCCAAATATAGCCTTTTTGTAAGACTTTCCCTCCCTTCATTGACATTGTATTTCAAACATTCAGCAACCCCTCCTGCAGA
ATCGTGTCCAGATGCATTCCTGATTAAATGCCACCGTAGTGCTCCCACGAACCGTTGGCAAGAGGAATTGCCAGGAACCTTTGGCTTAA
TTTCCAGCGATCCTAGCATCAGCTCGTCTGTGGTACAGTTCGAGGAATCAGCTGATGGCTTCATTGTTGGGAAGAAGTGATTAGAATCA
ATAAGGTTTTCCATATGTTTGCCCGGATGACTTGAGCAATCATGTCCTCTTTGGATGCTCCTCAAACGACAGCATTCAGCCTCAAGTGC
ATTCATTTTTTCTTCCATTTTTTTGATATCAGTGTCCAGCACTGAAATTTTCCTGGATTTTAAAGGGTAGGTGCAACTATTGCCATTGC
TAGATATTATACATGCTCTGCACGAAGGTCGATCCCCTGAACACCGTACTTTGCGACAGCTACATCGCGAGCATGCTACTTTGGAGCGC
TTTCTAGCTTTGTACATCTATTTCCACTACAAGAATTGTGCTCTTAAGTTAAATCAGATCCTTTGAGACCTATTTTGCAACAACTGGTG
TTTTTCCATGCCATTTGCTTTTTCAGCTAGCTTATCTCTACCGAGAAGGGAAACCGCGCCTGGGGAGCTCGCCGAGTCTCTTATCATCT
ATACAAACACATTATCGATGCAAGCAGGCAATGGAATTCTTGGTTTAATTTCTAGCAAAAGGATTCTGCGATTCAAACAGACATATGAG
CTCAATGAATAAATTATGAAAGAAATATTCCCTTCAAATTAGGGCTGTATATTAGCCAATTTCCATTACTCATGATAATTGATAAGCAT
ATATGAACGTAATGAAATCGATTGGCAAATATGTGAAAAGGGGACCGTATTTTGGGCGTGCTGATATGCAATATTAAAACTTGTAATAA
TTAAGGACCCCACTAATTAGCTGTACCAAAAGGAAGTACTCTATGGGCTAACCTTTTCACTCCTTACAGGGGATTGTGGTTTTGGACCG
TTAATAATAGAAGATAATTCATCAATTGGAAAAGTTGTCATTTCATTTCTATAAGATCAATATTCCGAATCAAAAACGTTTGATTATTA
ACACTAACATTCTTAGCTCCACAAATTTGAATGTTAATGGTGAAAAGAAAAAATGCAGCAGCCAGAATTATGGCATTCAAAAATGGTCC
TCGGAGAGATAGCAGAATCAAGTTATATTTATTTGGGTAGAATATCCCAATCAGAGAGCCTGCTGAACGGCTTTACTCAAAGATTAAAA
ATCCGAACGTTTTAACAATGTTTACAAGTACGTTTTTCTCTGTATTTCATAGACAAAAAAAAAAACACAAACTGAGACTTGTTTATCGG
ATTGTAGTTACAATTTATCAAGTGAACTTACGATTGAGAGGATTCGGTTCGTAATTTTGTGCAGAGAAGGATAAAAATAGTAATTTCCC
AGCAATGGAACTTTTATTTAATGGCAACTAGCAACTAAAGTGTGAATATTAGGTTATGCACAATTCATCAGTGATGCTGGATAATTATC
AATTTGCTTGAAATATGTTATTGGGACAATTGTTTTTGAAACACTGTTGCGACATACTGGTGGTGCTCGGCATTATATTGCACTTGATG
CCGAGTTTTAATGATCCTTCTCGGCAATATCATAATTTTACAGACAGCATTTAAGAAACATACTCGCGGGAGAATGTGCAAATTACGCC
AATAATAGAATATTAGCTTTAATCAAAGGTTAGTTTAGGAATGCGCTCGAATAGCGGACGAGTATCCGCAGTTTCATTGAAAAATATCG
ACTCGAGTCTTGATAAGAAATGATTTTATGACTTTCAAAGCAGCTTGGCACTATTTTGAAGTCAATGCCTCTCCATGTATACAATACTT
TGACCCTAGTTGCACCACGTATATCATCTGCTCATTGGCTCCACACCCTATTCAACTCCGGTAATGCAATTATTTGTAAGCATTGCAAT
GAAGATCTTTTTGAGGTAATATTAAACTCATGCAACCAGAAGTGGTCTGACGCATCCACCAATAGTTTTGGAAAATCTTTAGATTTGTA
TTCAAGGCTGCTCATCTCTCCCTGCATATGAAAGTTTATTTTTTGGCAGGGACTTAGACGGACCCTCAGGGTAAAGCTGGTTAAATTAG
TGAATCATCGTGTACGAAGACAATTGAAAGCCACCATTGTGGCTCACGTGGGTTGAAGAAGTCTTTGGAGTGTTGATAATATTTGCTCC
CAGTGCGATAGCGGGAACATGAAGATGATTAACCGGACTAGGTGAAATTGTGGCGACTGTGGAAGTCACACCTAATCTCAGTGCTTGCC
AAGTGCTGCACGGCAGAGTTTGACAGGCACTTGGCAGGGTTTAAAATTTGCAGATCTATGGTATAAGAACAGCTCAGGATGTTGTGGGA
TAATTTCACATTCAAATATTCTATGCCGAATTTATGATTATATATAAGAAGTTTCTATTATTGTTCTAACATGATCTCTAAAAGAATTG
AGTTCAAAGCTGCAGCTGCAAACTAGATATGTCCTCTTTGGCTAAAGAATTATTAGTGGACCCTTGGCGCAGATTGAAATGGGGGTTTA
TTCCGGTGAGAAGAGAGGTTACAGATCTTGATGCTGGTGAAGCAAAGGAACTGGTCCCATCAGAAGTCGAAAAAGAAGAAGGTACGTCC
TTAGAATTATCTGGAGACCGCACAAGTTCCGCTGATGGTGAACTTACCAAAGAAGAGTTCAGGGAGACCAAAAAAAACTTGGATACGGC
AAGCACAGATATTTCCGAGTATGAATACAAAGATGAAGCTAATAGGAAATGGTACAAATTTTTTGACGAGCAAGAGTATCGTATCAAAA
AATCCCAAGCTTCCAATAATAAATGGTACAGCTGGTTCAATGCAGGAACATCAACTAAAGAAAAAAAATTGTTGTTGAAGCTTGATGTG
TTACTAGCATTTTATTCATGTCTGGCCTACTGGGTTAAATACTTGGATACTGTTAACTTGAATAACGCATACGTTTCTGGTATGCAAGA
GGAGTTGAAGTTCAAAGGAAATGATCTTGTTCACACTCAAGTGATGTTCACTGTGGGCAATATTATTTTTCAAATTCCATTCCTTTTCT
TATTGAATAGAGTTCCATTAAATTATCTCTTACCAGTACTAGATTTGTGCTGGTCGCTATTAACCGTAGGAGAGGCATATGTCAACAAC
GTTCCTCATTTAAAGGCTATAAGATTTTTTGTTGGAGCATTCGAGGCACCAGCTTATCTGGCATATCAATATTTATTTGGTTGTTTCTA
CAAGCATGATGAAATGGTTCGTCGTTCCGCATTTTATTATTTGGGTCAATACATAGGTATTCTTTCGGCTGGTGGGATTCAATCGGCCG
TGTATTCTTCCCTGAACGGTCGAAATGGAATGTCAGGCTGGAGATGGAATTTTATCATTGATGCTATAATATCTGTAGCAGTTGGTATT
ATCGGATTCTATAGTTTGCCTGGTGATCCTTACAATTGCTATTCCATCTTTCTCACGGATGATGAAATTAGATTGGCTAGAAAAAGATT
GAAAGAGAATAACACCGAAAAATCTGACTTCTCTGCAAAAATATTTGATATCAAAGTTTGGAAAAGAATTATATTTGATTGGAAAATAT
ACGTCTTGACTTTGTGGAATATCTTTTGCTGGAACAACAATAATGTGTCATCTGGTGCTTATTTACTGTGGTTGAAATCTCTAAAACGT
TATTCAGTTCCAAAGTTAAATCAACTATCTATGATAACCCCTGGTTTAGGAATGGTTTATTTAGCTGTGACTGGTTTACTGGCCGACAA
ATCCCATTCTCGTTGGTTTGCAATTGTATTTACGCAGCTTTTTAATATAACAGGAAACGTTATACTTGCAGTATGGGACGTCAGCGAGG
GAGGTAAGTGGTTCGCCTTCATGTTGCAATGTTTTGGTTGGGCCATGGCACCTGTTCTATATTCTTGGCAAAATGACATATGCCGTAAA
GATTCGCAAGCACGGGCGGTCACTTTGGTGGTCATGAATATGCTTGCCCAAACTAGCACGGCATTTATTAGTGTCTTAGTGTGGAAAAC
AACGGAGGCTCCGAGGTATCTAAAAGGTTTCACTTGGACAGCCTGCTCAAGTTTTTGCTTAACTATCTGGACTTTTGTCGTTCTGTACT
TTTACAAACGTGAAGAGAGAAAAAAATGCGTTAGATAATGGAATTATACTCTATAATTCAGCCAATGGTGAAAGCCCTTCAATTTCGCAC
GATATAGAGGATCCAGAGAATTACTAAATATTTGATATATCTTTAATACAAGTAGTGAACTCATAAAGATTCTTTTCATAGATTTTATG
TAGTTTTAGTATTTTTTTTGTTTAATATTTTACTTTGTTAGGGTCCCTTAAATGCGCAGCGGGCCTATCCCCAAGTTAAATTTTAAGTT
TTGTGAGCATCAATTGTAATTCAAAGGTAATGATTGTAGAAGAAAATCCTTATGAATGCAATAAGAGAAATTAAATTCATAGCGAGAAA
TATGAAGTCATATATGATGTGAAATGAAGGAACGACTTGAATATGTGGTCGATTCGGGTCGGTTTAGATGAGTGGGCTAGTAACGATCC
AAGCACTACCCACAACGCTTTCAAGGCAGTTATCTGAAACATTGTTAGATGGGGTGGAAGGGTCATTAAATTAAAACAAAATAAGCGTT
ACAGTAGCAATTGGGTACAGGGAGTAATCCGTGGTGCTACATCCCCATGAGGTAGGTTTCTGTAAGTGAATGTTGCTATATTTATGCCA
TATTGTCTACGTGAACTTGTTCTTGAATTGAACTAAGGGCAAGTACTAGCCAGTCATTCGGAAATAATGATGTAATAATTCCTCAGGAT
TCCAGCCTCCTAACCTTGGATGACTCATTTGAAGAAACCGGCTATTTATTTGATCCGCCCATTACAGCTTGAAAATACTGGTGGTGGCG
CCCCAGTTGATATGCTATAAATGTCTAGTGAGCAGTGAAGAAATGGCCGCTATATGCGACGAAGGACATCAAGATGTGAGAAGAGAAAG
AGCGATGGCGTAACTGCTGTCATTGGTGGTTATCGTTTGACAGACTATTTAGAGAAGTCCAAATTCAAAAGGAATCTTGGGTAAGGAGA
GCCGGCGGTTGTTGAGGTTCCGAGTCAATGGTTGCATCACCAACAGCGCTACGAATCACAGTGCACGACATGTCGGTCCGTTGTTACAG
CGATTGTACAATTGCCAAACCACATTTGGGCCAGAACCGGATAAACCCCGTGAAACAAAAAGCCGAAAATTGCAAGATGCATCTCATCC
AGGGAAGGTGGTAAAACCGCAAGAAAGCTTTCGTGCTAAGATCTTTCCAATTTTAAATTGGATCAGTCTGCATGCCAGGCGATGTCTGA
TTTAGTTAACTTGCGATTGTTATGCCATTCGCTATACCTTTATCACCGAATTTGTGAAAGAGAGGTCAAAATATACTATTCTGGGGAGC

CAAAAGAATAGTAAAAGAAATTGAAATAACTGAATATAGCCAGATTATAAAAGCCGATTCCTTAGCTCATCCCATATCCCTTCCCACAC
CTGCGATTGCCTGTTCTATCAATTTTTCAGGTAGAGTATCTCAGTTTCACTTTAACGCTGCGACGGAAATCCCAGCTGTTACTAGACAC
ACCAATGTGATCCGCCCCTTCAAGATGATTGATGTGGAGCACTCTCCTAAGCTAAAAAGAACCAATGGAAACTCCCGGGGAGCGTGGTA
GACAGATTTTCAATTTAAGAGAGCATGCAGATGGTGCTCCCCTGTCGTGAAGATAGAAAATCCACAGCCAGTAGTTGGAGAAGAGTTGG
CGGTCTTGCCGAGTAGTTGCAAGTCGGAGACTCTTAGAAAACCTTCGAGATCTGCGTGTAAGTGAAAATGAGGAAGAAGCTCGAATATT
ATAATTATCAAGATTCGAATGGCCTGCACACTTTGGGAAACTCTTCACTCTTACTCTTGGAAAAATTCATCTAGGGGTCTGTAGTAGTT
GATTGTGAAGTGGTACCAACAATGTCGCCAAAATAGATAAAGAGGTAATAATGCAATATACTGTCCCGCTAGGACTATCAGCCACTAAT
GAAGACCCTTCCTTCGTGATTAGAATTCAAGCCTTCAAAGCTTACTTTTTTTTGGCTACCTCTTCTAAATTAATGGATATTCAGGAAA
TGCACGATTGAAAAGTGACCATCGAAGATGCAGACACGAAGGCCAGGATTTGTTTTTTTCTTGAATGTTCATGTGCGTGTGGCTCTAA
AATGCGATGTCTCCTGTCTTCGTAATATAGGAAAATCTTGGCCCTTCATTATTCTTCTCAAGTCTCTGACGCGATCATCTTCTATTGGT
ATCCTAAATTTTTATGATGTTTTATTGAAGCCCAGCTTCGATTTCCCTGTCCGAAATAATTCTTAGCAAAATGCACTAATCAATGGGGA
TGAAGAAAAACAATAGTTCTTGTAGGTTAATAGCAAATGAGTGGTAGCTGATGACGTTTTTCTTTTCAATAGTCCTTAAAGTGCGACCA
ACTCAAACTCCTGTGGAAATTGTTTAGGAATTTGAAGGTGTATCAAAATGAACGAATTGGTGAATTAGCATTTTAAATTTTTCAAGTGG
AATTTGAAAAAGGTGGAATAGAAATCGATACATTTTCCATATAGGTTAATTTATAATTAAACGAGCAAAACATAACCTACTTTATACAT
ATTAGTTATATTTTATAGATCTGCTTGCCATCTTGCACAATATATCCATTCAAGTCCCTCACCATGGGATTTCCGTCGTCCTGTGATGG
CAACTGCCTTAGGCCACAGGAGAAGTGCAGGTGAATGGTTGCGCAGGGTTGGTCTGATAGACTGCGATAGCTCCCGAAATTGCCGAGTA
GGAACATAGGCCACAAGTTGCGAGCGAGGCTAGGAAAAGCAAAAAGAGCGAAACTTTAATCGAAGTGCGGTAAATGCCTCCATACCCTA
AAGCACGCTTCCACCTAGACCACTCGCGATATGAGTCAGCTTTCTCCACAGTTAGAGTCTTAGCATCGAAGTTGTCGGCCAATGCCGCA
TCTTGGCGGCATAACAGGCAGAATCCAAAAAGAAATGGGAATGTGTACGAGAAATTCAGGATACAGAAAGCACCGACGATCCCAACCAA
TGCACTGATAGATGGAATGGCTGTACCAAGAATATAAGCGACAGCCCAGTACAGAATAACAAATCCTCCCCATGCGAATGTTCCCTTGC
GCGAGGTAAGGCTGGGGAAGTTAAAATCTGTTACCAGAAAACCCTGGTAAACAACTTTAATACCAATATTACCGTAAAGCACCATAGCA
ATTATACCAGATATAATGGAAAGCACATTGCCAGCATTCTGCAATCCTATGGAGCCAATGGCCATGTTTGCAGTCACATAACTAAATTG
TCCATTGTAAGCATACACGAAAAGTCCGTAGAAAAGATAAACGACCAAAATTAGAGATTGCGCACAGAGCATACCTTTCCAAAAATCCA
TTGGACGCTTCATTTCTGCCATAACCTCGCAGAAAATCGTGGCACCACCCCAAGCAAAAACCATATTGTTCATACCTGCAATTTTATCA
CTAATATTACCAGGCACGATGGCGTAGTGTTTAATTGGCAATGGGATGTAACTACTAGGTTGGAAGTAAGGGGCGCCTTGCCCATATTG
TTCAAAAATACCGTAGTAAGGACCCCCCACAGCAACACCTACCATTGTGATAATACAGATTGCGATGTTCATCCACACGGCAGAGTTTG
CAATATGTGCAAATCTGCCGAGAGAACGAATCTGGCCCATGATCATACATAGGAGACCCCACACTAAAATGTCAACGGTAAAGCAGAAG
TGATCTCCGCCTCTGTTTACAACTAGCATCTGTGACACAGATTGAGCGGTGCTTAAAAGTAGTAATCCACAATTTAGTATCATTTGAAT
AAACTGTAGAAGCCCGAACGGATACCGGAACCAAGGCACAACAACACGCGCAGCCAGGTCAGAAAATGTACGAATTGGATAGTTGTTAG
AGTCGACTTTGCAAAAGCAGAAGTTCAACAACCAACCGCCAAACGCAGCAGCGACACCGAAAATGACATAAAGGATAACTCCTGGTACC
CATCCCATTTGGGCAACAGCATATGGAGCATTAGATGGGCCCAGAATATCGGTAGTGATTAAAAAGAACACGGAATATGCCTTAGCTAT
GCGCAGCATTCTCTGTGAATCAAGCTTCGATGGTGAGACATTGCTGTGAGGGTCTGTATACATCTGTTCTCTCTGAACAGCATACAGGC
CATCTTCATCTCTTTCTCTCTCGAATTCACGCTGGATCTCAGCGTAGTGAACATAAGCCTGCAGTGGTAGTTGGCGTCCATGCTTCTCG
CTCTCACTGGAAATGATCTCACGGCCGGATATGTCAACATATTCTCCTTTCTCATGATCAATGGTCCCTTGAGATCCTTTTTCTTTCTT
CGCCTCATCGTAGGCCCTCGATGTCATCATCGATAGGTTAGGACTAGCGATAGGGACAAGTTTTGACATCTTCAGCGATAAATATTTTT
TTTTTCAGTCGAATGGAACGTCGTATTGTCGTTACGGAAAAATTATAGTTTCGAAATTGCGATTAAGCTTATTATTGTGTATATTGAGG
ATGCTACACTGCTGTTTAGTTCTTGTATATTTGTGACTAGTTTTAATGGAATAATGCTTATGTATATACGATTATTGACACATTTTATA
CTGACACCTTTCATCGTTTTTTTTTTTGTCTTGGCTGTATCTTCTCGTTATCTTGTCCTTTTTTGACAGCATCCTTCACAGTGTCTTGT
TTTCTTCTTGGCTGCCCCACTTCCTTAGATATCGTGTGCTCATTATCGCTTTTACCAAGTTGTAATCTAGAAAGATAACAGATAGTCAA
CGAGACTGAGAGTTCGCAAAATCGCACTCAAAAGCCCCTAGGAAGGAAAACAAACATCACAGACCTGCAACCTTTGAAATTCTACATCT
ATCAAAGATAAGATTCGACACTTTTTTAGAAAAGCGATTTTGACAGCATCATGCGGTTTTTACAACTTTTTAGTAAAATGTAGCAATCT
TATCCGCTTTTCCGTGACACATTTTCTCAGTTTTCCACAGTTTGATTCTCGAGCACTGGAAAGATAAAAAAGAAAGGACGATCTCGGGG
CACGATACCAAAAATTCCGATGAGTGAGAAATTTCTGAAAAATGGTGCCGTGTGGATGTGTCAAGCGAGATTGTGCCAAGAAAATGACA
TGATGAGATCCCGCCCGGAATAAACCTAGCAAGTTGATATCAATATTTTTTTAATGTTACTTATCTTCATTGCTAATAATTCGTGAAA
TGATTGAAAATTTCCAATCCA
[matches with chr XV from 1,054,629 to 1,067,195]
[matches with *ARR* genes cluster from 1483 to 1575]

>SGU90_unitig1536
TACAGTATTACTGATGATATGTTATTTAGTGCTGTCCGTATGTCCTCCTGTTCTTTTTTGGGCTACAATTTAATTGATGGGGTGTTATG
CTTACGATATTTCAATTATGTTAATATTTAGTATGTTTATATCTTCTCATTAATGAATGGTTAATTTTTA
[matches with chr VIII from 555989 to 556053]
[matches with *ARR* genes cluster from 16,636 to 16,730]

>SGU90_unitig1784
TCACCTAAACGTAAAAATTAACTATTCATTAATGAGAAGATATAAACATACTAAATATTAACATAATTGAAATATCGTAAACATAACAC
TCCATCAATTAAATTGTAGCCCAAAAAAGAACAGGAGGGCATACGTACAGCACTAAATAACATATCATCAGTAATATTGTATTGATGAT
GCCATTTTATCATTCAGTTATGTAAACGCTTTTCGTAAGAATGTAAATTATGAACGCCACCTTCTAGTTGAGCAGAAGCGGTTCTGAAT
TCAAATCTAA
[matches with chr VIII from 556161 to 555989]
[matches with *ARR* genes cluster from 16,636 to 16,741]

>SGU90_unitig4710
TACAGTATTACTGATGATATGTTATTTAGTGCTGTCCGTATGTCCTCCTGTTCTTTTTTGGGCTACAATTTAATTGATGGGGTGTTATG
TTTACGATATTTCAATTATGTTAATATTTAGTATGTTTATATCTTCTCATTAATGAATAGTTAATTTTTACGTTTAGGTGA
[matches with chr VIII from 555989 to 556053]
[matches with *ARR* genes cluster from 16,636 to 16,741]

>SGU90_unitig1284
TGGATTGGAAATTTTCAATCATTTCACGAATTATTAGCAATGAAGATAAGTAACATTAAAAAAAATATTGATATCAACTTGCTAGGTTT
ATTCCGGGCGGGATCTCATCATGTCATTTTCTTGGCACAATCTCGCTTGACACATCCACACGGCACCATTTTTCAGAAATTTCTCACTC
ATCGGAATTTTTGGTATCGTGCCCCGAGATCGTCCTTTCTTTTTTATCTTTCCAGTGCTCGAGAATCAAACTGTGGAAAACTGAGAAAA
TGTGTCACGGAAAAGCGGATAAGATTGCTACATTTTACTAAAAAGTTGTAAAAACCGCATGATGCTGTCAAAATCGCTTTTCTAAAAAA
GTGTCGAATCTTATCTTTGATAGATGTAGAATTTCAAAGGTTGCAGGTCTGTGATGTTTGTTTTCCTTCCTAGGGGCTTTTGAGTGCGA
TTTTGCGAACTCTCAGTCTCGTTGACTATCTGTTATCTTTCTAGATTACAACTTGGTAAAAGCGATAATGAGCACACGATATCTAAGGA
AGTGGGGCAGCCAAGAAGAAAACAAGACACTGTGAAGGATGCTGTCAAAAAAGGACAAGATAACGAGAAGATACAGCCAAGACAAAAAA
AAAAACGATGAAAGGTGTCAGTATAAAATGTGTCAATAATCGTATATACATAAGCATTATTCCATTAAAACTAGTCACAAATATACAAG
AACTAAACAGCAGTGTAGCATCCTCAATATACACAATAATAAGCTTAATCGCAATTTCGAAACTATAATTTTTCCGTAACGACAATACG
ACGTTCCATTCGACTGAAAAAAAAAATATTTATCGCTGAAGATGTCAAAACTTGTCCCTATCGCTAGTCCTAACCTATCGATGATGACA
TCGAGGGCCTACGATGAGGCGAAGAAAGAAAAAGGATCTCAAGGGACCATTGATCATGAGAAAGGAGAATATGTTGACATATCCGGCCG
TGAGATCATTTCCAGTGAGAGCGAGAAGCATGGACGCCAACTACCACTGCAGGCTTATGTTCACTACGCTGAGATCCAGCGTGAATTCG
AGAGAGAAAGAGATGAAGATGGCCTGTATGCTGTTCAGAGAGAACAGATGTATACAGACCCTCACAGCAATGTCTCACCATCGAAGCTT
GATTCACAGAGAATGCTGCGCATAGCTAAGGCATATTCCGTGTTCTTTTTAATCACTACCGATATTCTGGGCCCATCTAATGCTCCATA
TGCTGTTGCCCAAATGGGATGGGTACCAGGAGTTATCCTTTATGTCATTTTCGGTGTCGCTGCTGCGTTTGGCGGTTGGTTGTTGAACT
TCTGCTTTTGCAAAGTCGACTCTAACAACTATCCAATTCGTACATTTTCTGACCTGGCTGCGCGTGTTGTTGTGCCTTGGTTCCGGTAT
CCGTTCGGGCTTCTACAGTTTATTCAAATGATACTAAATTGTGGATTACTACTTTTAAGCACCGCTCAATCTGTGTCACAGATGCTAGT
TGTAAACAGAGGCGGAGATCACTTCTGCTTTACCGTTGACATTTTAGTGTGGGGTCTCCTATGTATGATCATGGGCCAGATTCGTTCTC
TCGGCAGATTTGCACATATTGCAAACTCTGCCGTGTGGATGAACATCGCAATCTGTATTATCACAATGGTAGGTGTTGCTGTGGGGGGT
CCTTACTACGGTATTTTTGAACAATATGGGCAAGGCGCCCCTTACTTCCAACCTAGTAGTTACATCCCATTGCCAATTAAACACTACGC
CATCGTGCCTGGTAATATTAGTGATAAAATTGCAGGTATGAACAATATGGTTTTTGCTTGGGGTGGTGCCACGATTTTCTGCGAGGTTA
TGGCAGAAATGAAGCGTCCAATGGATTTTTGGAAAGGTATGCTCTGTGCGCAATCTCTAATTTTGGTCGTTTATCTTTTCTACGGACTT
TTCGTGTATGCTTACAATGGACAATTTAGTTATGTGACTGCAAACATGGCCATTGGCTCCATAGGATTGCAGAATGCTGGCAATGTGCT
TTCCATTATATCTGGTATAATTGCTATGGTGCTTTACGGTAATATTGGTATTAAAGTTGTTTACCAGGGTTTTCTGGTAACAGATTTTA
ACTTCCCCAGCCTTACCTCGCGCAAGGGAACATTCGCATGGGGAGGATTTGTTATTCTGTACTGGGCTGTCGCTTATATTCTTGGTACA
GCCATTCCATCTATCAGTGCATTGGTTGGGATCGTCGGTGCTTTCTGTATCCTGAATTTCTCGTACACATTCCCATTTCTTTTTGGATT
CTGCCTGTTATGCCGCCAAGATGCGGCATTGGCCGACAACTTCGATGCTAAGACTCTAACTGTGGAGAAAGCTGACTCATATCGCGAGT
GGTCTAGGTGGAAGCGTGCTTTAGGGTATGGAGGCATTTACCGCACTTCGATTAAAGTTTCGCTCTTTTTGCTTTTCCTAGCCTCGCTC
GCAACTTGTGGCCTATGTTCCTACTCGGCAATTTCGGGAGCTATCGCAGTCTATCAGACCAACCCTGCGCAACCATTCACCTGCACTTC
TCCTGTGGCCTAAGGCAGTTGCCATCACAGGACGACGGAAATCCCATGGTGAGGGACTTGAATGGATATATTGTGCAAGATGGCAAGCA
GATCTATAAAATATAACTAATATGTATAAAGTAGGTTATGTTTTGCTCGTTTAATTATAAATTAACCTATATGGAAAATGTATCGATTT
CTATTCCACCTTTTTCAAATTCCACTTGAAAAATTTAAAATGCTAATTCACCAATTCGTTCATTTTGATACACCTTCAAATTCCTAAAC
AATTTCCACAGGAGTTTGAGTTGGTCGCACTTTAAGGACTATTGAAAAGAAAAACGTCATCAGCTACCACTCATTTGCTATTAACCTAC
AAGAACTATTGTTTTTCTTCATCCCCATTGATTAGTGCATTTTGCTAAGAATTATTTCGGACAGGGAAATCGAAGCTGGGCTTCAATAA
AACATCATAAAAATTTAGGATACCAATAGAAGATGATCGCGTCAGAGACTTGAGAAGAATAATGAAGGGCCAAGATTTTCCTATATTAC
GAAGACAGGAGACATCGCATTTTAGAGCCACACGCACATGAACATTCAAGAAAAAAAAACAAATCCTGGCCTTCGTGTCTGCATCTTCGA
TGGTCACTTTTCAATCGTGCATTTCCTGAATATCCATTAATTTAGAAGAGGTAGCCAAAAAAAAAAGTAAGCTTTGAAGGCTTGAATTCT
AATCACGAAGGAAGGGTCTTCATTAGTGGCTGATAGTCCTAGCGGGACAGTATATTGCATTATTACCTCTTTATCTATTTTGGCGACAT
TGTTGGTACCACTTCACAATCAACTACTACAGACCCCTAGATGAATTTTTCCAAGAGTAAGAGTGAAGAGTTTCCCAAAGTGTGCAGGC
CATTCGAATCTTGATAATTATAATATTCGAGCTTCTTCCTCATTTTCACTTACACGCAGATCTCGAAGGTTTTCTAAGAGTCTCCGACT
TGCAACTACTCGGCAAGACCGCCAACTCTTCTCCAACTACTGGCTGTGGATTTTCTATCTTCACGACAGGGGAGCACCATCTGCATGCT
CTCTTAAATTGAAAATCTGTCTACCACGCTCCCCGGGAGTTTCCATTGGTTCTTTTTAGCTTAGGAGAGTGCTCCACATCAATCATCTT
GAAGGGGCGGATCACATTGGTGTGTCTAGTAACAGCTGGGATTTCCGTCGCAGCGTTAAAGTGAAACTGAGATACTCTACCTGAAAAAT
TGATAGAACAGGCAATCGCAGGTGTGGGAAGGGATATGGGATGAGCTAAGGAATCGGCTTTTATAATCTGGCTATATTCAGTTATTTCA
ATTTCTTTTACTATTCTTTTGGCTCCCCAGAATAGTATATTTTGACCTCTCTTTCACAAATTCGGTGATAAAGGTATAGCGAATGGCAT
AACAATCGCAAGTTAACTAAATCAGACATCGCCTGGCATGCAGACTGATCCAATTTAAAATTGGAAAGATCTTAGCACGAAAGCTTTCT
TGCGGTTTTACCACCTTCCCTGGATGAGATGCATCTTGCAATTTTCGGCTTTTTGTTTCACGGGGTTTATCCGGTTCTGGCCCAAATGT
GGTTTGGCAATTGTACAATCGCTGTAACAACGGACCGACATGTCGTGCACTGTGATTCGTAGCGCTGTTGGTGATGCAACCATTGACTC
GGAACCTCAACAACCGCCGGCTCTCCTTACCCAAGATTCCTTTTGAATTTGGACTTCTCTAAATAGTCTGTCAAACGATAACCACCAAT
GACAGCAGTTACGCCATCGCTCTTTCTCTTCTCACATCTTGATGTCCTTCGTCGCATATAGCGGCCATTTCTTCACTGCTCACTAGACA
TTTATAGCATATCAACTGGGGCGCCACCACCAGTATTTTCAAGCTGTAATGGGCGGATCAAATAAATAGCCGGTTTCTTCAAATGAGTC
ATCCAAGGTTAGGAGGCTGGAATCCTGAGGAATTATTACATCATTATTTCCGAATGACTGGCTAGTACTTGCCCTTAGTTCAATTCAAG
AACAAGTTCACGTAGACAATATGGCATAAATATAGCAACATTCACTTACAGAAACCTACCTCATGGGGATGTAGCACCACGGATTACTC
CCTGTACCCAATTGCTACTGTAACGCTTATTTTGTTTTAATTTAATGACCCTTCCACCCCATCTAACAATGTTTCAGATAACTGCCTTG
AAAGCGTTGTGGGTAGTGCTTGGATCGTTACTAGCCCACTCATCTAAACCGACCCGAATCGACCACATATTCAAGTCGTTCCTTCATTT
CACATCATATATGACTTCATATTTCTCGCTATGAATTTAATTTCTCTTATTGCATTCATAAGGATTTTCTTCTACAATCATTACCTTTG
AATTACAATTGATGCTCACAAAACTTAAAATTTAACTTGGGGATAGGCCCGCTGCGCATTTAAGGGACCCTAACAAAGTAAAATATTAA
ACAAAAAAAATACTAAAACTACATAAAATCTATGAAAAGAATCTTTATGAGTTCACTACTTGTATTAAAGATATATCAAATATTTAGTA
ATTCTCTGGATCCTCTATATCGTGCGAAATTGAAGGGCTTTCACCATTGGCTGAATTATAGAGTATAATTCCATTATCTAACGCATTTT
TTCTCTCTTCACGTTTGTAAAAGTACAGAACGACAAAAGTCCAGATAGTTAAGCAAAAACTTGAGCAGGCTGTCCAAGTGAAACCTTTT
AGATACCTCGGAGCCTCCGTTGTTTTCCACACTAAGACACTAATAAATGCCGTGCTAGTTTGGGCAAGCATATTCATGACCACCAAAGT
GACCGCCCGTGCTTGCGAATCTTTACGGCATATGTCATTTTGCCAAGAATATAGAACAGGTGCCATGGCCCAACCAAAACATTGCAACA
TGAAGGCGAACCACTTACCTCCCTCGCTGACGTCCCATACTGCAAGTATAACGTTTCCTGTTATATTAAAAGCTGCGTAAATACAATT
GCAAACCAACGAGAATGGGATTTGTCGGCCAGTAAACCAGTCACAGCTAAATAAACCATTCCTAAACCAGGGGTTATCATAGATAGTTG
ATTTAACTTTGGAACTGAATAACGTTTTAGAGATTTCAACCACAGTAAATAAGCACCAGATGACACATTATTGTTGTTCCAGCAAAAGA

TATTCCACAAAGTCAAGACGTATATTTTCCAATCAAATATAATTCTTTTCCAAACTTTGATATCAAATATTTTTGCAGAGAAGTCAGAT
TTTTCGGTGTTATTCTCTTTCAATCTTTTTCTAGCCAATCTAATTTCATCATCCGTGAGAAAGATGGAATAGCAATTGTAAGGATCACC
AGGCAAACTATAGAATCCGATAATACCAACTGCTACAGATATTATAGCATCAATGATAAAATTCCATCTCCAGCCTGACATTCCATTTC
GACCGTTCAGGGAAGAATACACGGCCGATTGAATCCCACCAGCCGAAAGAATACCTATGTATTGACCCAAATAATAAAATGCGGAACGA
CGAACCATTTCATCATGCTTGTAGAAACAACCAAATAAATATTGATATGCCAGATAAGCTGGTGCCTCGAATGCTCCAACAAAAAATCT
TATAGCCTTTAAATGAGGAACGTTGTTGACATATGCCTCTCCTACGGTTAATAGCGACCAGCACAAATCTAGTACTGGTAAGAGATAAT
TTAATGGAACTCTATTCAATAAGAAAAGGAATGGAATTTGAAAAATAATATTGCCCACAGTGAACATCACTTGAGTGTGAACAAGATCA
TTTCCTTTGAACTTCAACTCCTCTTGCATACCAGAAACGTATGCGTTATTCAAGTTAACAGTATCCAAGTATTTAACCCAGTAGGCCAG
ACATGAATAAAATGCTAGTAACACATCAAGCTTCAACAACAATTTTTTTTCTTTAGTTGATGTTCCTGCATTGAACCAGCTGTACCATT
TATTATTGGAAGCTTGGGATTTTTTGATACGATACTCTTGCTCGTCAAAAAATTTGTACCATTTCCTATTAGCTTCATCTTTGTATTCA
TACTCGGAAATATCTGTGCTTGCCGTATCCAAGTTTTTTTTGGTCTCCCTGAACTCTTCTTTGGTAAGTTCACCATCAGCGGAACTTGT
GCGGTCTCCAGATAATTCTAAGGACGTACCTTCTTCTTTTTCGACTTCTGATGGGACCAGTTCCTTTGCTTCACCAGCATCAAGATCTG
TAACCTCTCTTCTCACCGGAATAAACCCCCATTTCAATCTGCGCCAAGGGTCCACTAATAATTCTTTAGCCAAAGAGGACATATCTAGT
TTGCAGCTGCAGCTTTGAACTCAATTCTTTTAGAGATCATGTTAGAACAATAATAGAAACTTCTTATATATAATCATAAATTCGGCATA
GAATATTTGAATGTGAAATTATCCCACAACATCCTGAGCTGTTCTTATACCATAGATCTGCAAATTTTAAACCCTGCCAAGTGCCTGTC
AAACTCTGCCGTGCAGCACTTGGCAAGCACTGAGATTAGGTGTGACTTCCACAGTCGCCACAATTTCACCTAGTCCGGTTAATCATCTT
CATGTTCCCGCTATCGCACTGGGAGCAAATATTATCAACACTCCAAAGACTTCTTCAACCCACGTGAGCCACAATGGTGGCTTTCAATT
GTCTTCGTACACGATGATTCACTAATTTAACCAGCTTTACCCTGAGGGTCCGTCTAAGTCCCTGCCAAAAAATAAACTTTCATATGCAG
GGAGAGATGAGCAGCCTTGAATACAAATCTAAAGATTTTCCAAAACTATTGGTGGATGCGTCAGACCACTTCTGGTTGCATGAGTTTAA
TATTACCTCAAAAAGATCTTCATTGCAATGCTTACAAATAATTGCATTACCGGAGTTGAATAGGGTGTGGAGCCAATGAGCAGATGATA
TACGTGGTGCAACTAGGGTCAAAGTATTGTATACATGGAGAGGCATTGACTTCAAAATAGTGCCAAGCTGCTTTGAAAGTCATAAAATC
ATTTCTTATCAAGACTCGAGTCGATATTTTTCAATGAAACTGCGGATACTCGTCCGCTATTCGAGCGCATTCCTAAACTAACCTTTGAT
TAAAGCTAATATTCTATTATTGGCGTAATTTGCACATTCTCCCGCGAGTATGTTTCTTAAATGCTGTCTGTAAAATTATGATATTGCCG
AGAAGGATCATTAAAACTCGGCATCAAGTGCAATATAATGCCGAGCACCACCAGTATGTCGCAACAGTGTTTCAAAAACAATTGTCCCA
ATAACATATTTCAAGCAAATTGATAATTATCCAGCATCACTGATGAATTGTGCATAACCTAATATTCACACTTTAGTTGCTAGTTGCCA
TTAAATAAAAGTTCCATTGCTGGGAAATTACTATTTTTATCCTTCTCTGCACAAAATTACGAACCGAATCCTCTCAATCGTAAGTTCAC
TTGATAAATTGTAACTACAATCCGATAAACAAGTCTCAGTTTGTGTTTTTTTTTTTGTCTATGAAATACAGAGAAAAACGTACTTGTAA
ACATTGTTAAAACGTTCGGATTTTTAATCTTTGAGTAAAGCCGTTCAGCAGGCTCTCTGATTGGGATATTCTACCCAAATAAATATAAC
TTGATTCTGCTATCTCTCCGAGGACCATTTTTGAATGCCATAATTCTGGCTGCTGCATTTTTTCTTTTCACCATTAACATTCAAATTTG
TGGAGCTAAGAATGTTAGTGTTAATAATCAAACGTTTTTGATTCGGAATATTGATCTTATAGAAATGAAATGACAACTTTTCCAATTGA
TGAATTATCTTCTATTATTAACGGTCCAAAACCACAATCCCCTGTAAGGAGTGAAAAGGTTAGCCCATAGAGTACTTCCTTTTGGTACA
GCTAATTAGTGGGGTCCTTAATTATTACAAGTTTTAATATTGCATATCAGCACGCCCAAAATACGGTCCCCTTTTCACATATTTGCCAA
TCGATTTCATTACGTTCATATATGCTTATCAATTATCATGAGTAATGGAAATTGGCTAATATACAGCCCTAATTTGAAGGGAATATTTC
TTTCATAATTTATTCATTGAGCTCATATGTCTGTTTGAATCGCAGAATCCTTTTGCTAGAAATTAAACCAAGAATTCCATTGCCTGCTT
GCATCGATAATGTGTTTGTATAGATGATAAGAGACTCGGCGAGCTCCCCAGGCGCGGTTTCCCTTCTCGGTAGAGATAAGCTAGCTGAA
AAAGCAAATGGCATGGAAAAACACCAGTTGTTGCAAAATAGGTCTCAAAGGATCTGATTTAACTTAAGAGCACAATTCTTGTAGTGGAA
ATAGATGTACAAAGCTAGAAAGCGCTCCAAAGTAGCATGCTCGCGATGTAGCTGTCGCAAAGTACGGTGTTCAGGGGATCGACCTTCGT
GCAGAGCATGTATAATATCTAGCAATGGCAATAGTTGCACCTACCCTTTAAAATCCAGGAAAATTTCAGTGCTGGACACTGATATCAAA
AAAATGGAAGAAAAAAATGAATGCACTTGAGGCTGAATGCTGTCGTTTGAGGAGCATCCAAAGAGGACATGATTGCTCAAGTCATCCGGG
CAAACATATGGAAAACCTTATTGATTCTAATCACTTCTTCCCAACAATGAAGCCATCAGCTGATTCCTCGAACTGTACCACAGACGAGC
TGATGCTAGGATCGCTGGAAATTAAGCCAAAGGTTCCTGGCAATTCCTCTTGCCAACGGTTCGTGGGAGCACTACGGTGGCATTTAATC
AGGAATGCATCTGGACACGATTCTGCAGGAGGGGTTGCTGAATGTTTGAAATACAATGTCAATGAAGGGAGGGAAAGTCTTACAAAAAG
GCTATATTTGGATGATCAAGAAAGAGGTGATAATCAGAAAGCTATCTTACCTGAAAGAGCATATGCATCAGAACTGATCCACAGAGTCT
ACCAGTTCTTTGCAAAAGAGTATGGTTTATTCTCAATCACTGAGTTCCATGTCCGACTTGAAGAAACCTATAGAGATGTCCAATCGCAA
GATCCTAGCTGGCTTGCATACCTCATGGTAACCTTTGCAGTAGGGGGAGCAGTATACAAACGATGCTGCAGGCGTTGGTATCAAAAAAGT
ACCTGGAAGAAGCTTTTTTTCGACAGCCCTCACATTATTCCATGAACCGATTGAAGAGCCCACCTTAGATACAGTCAGAACATTGCTTT
TGTTTGCTATATACTCTCAAGGATGGAATAGAGTAAATTCTGGTTTCACCTACACTGGGCTCGCCTTATCGACTGCTATAATGTTAGGG
ATGCATAGAGAAGTAAGCAATGTTGGACGACCGAAGGCAGAACAAGATGCAAGAAAGAAAGTATGGTGGACTGCATTTTTTATGGACAT
CCTATGGGCTACTAGACTCGGCTTGCCGCCTCATTTTAACGTGGATTATATGGACCTTGATGTACCAGATAAAAATATATCTAACGAGG
ACGGGTTTGATCCAAAAATTTTAATTGCGAACACGAAATTAGCTCTTCACATTGGCTCGGTAATGAAGAAAGTTTATGTCACTTCTGGG
GACAATGATTTCATAACAAACATAGTCGACAGTTTAAAGCAGTTAGAACTTTTTCGAGATAGTCTTGACCCAGAATTAAGGGTTTTGCC
TGATATTATTGAGTCAAATAGGTCCATAGCGGTTTTAACTTTGCGTTACCATCAAATAATCATAGTGACTGGTCGGCCGCTTTACCTTT
CACTGTTGAAATCTACTATAAGAGTTACTGATGAACTTCAGGATGCCAAAGTCAAATGTGTTCTCGCTGCTGTAACAAATATCTGTATT
TTGAACAACCTTTGGAATTCTGGTTGGTTTTGCATTTTTGGGTTCCTCGAAGCGCAATGCTGCTTTTCATCCATTTTGATGCTGGTTAT
GGAAACATTGAATGGGGACGCGTTTCCTGAGCTACAGATAGCGTTGTCCTTGAATGCCTCAATGTGTAAAGCCGGCAATATTACGGCAT
TGGATAATTACAGTCGGCTGAAGGAACTCGATAGTATTCTGTTCGAGGCAGAAAAGGAGAGACAGAACAGGGAAGAATCTTCGATGAAA
TCTTTACAATCTAACCGCACCACAATTACTGAAGACGGTGAAAATTCCCTAGTAGAAAATGAATCACTGCATGCAAAAAAAAATTTCAAC
TCTTCAAGATAATGCTCCACTTTCTGAAGAACTGCGTCAGGACGAACTTAATGCGAATTTGCCATCCTTAATTGAATTAGAAAGCTCTG
GTGCAGGATTTGAACATTTTTCACCAGAGACCTTTCGTAACTTATCTATGAACTTTGAAAGGTGGGATGCTTCCCTCGATTTGTCAACA
GGAAGTTCTTTGTAGTAATTATTGATTCATAAAAGTGCAATTTTTTTCACTTCTCATCCTTCAGAATAGCAAAGCCCGCAATATGCCTA
TAAGGCCGTCTGCAACTACTGTGATTATTTAAGCCTGCAAAAAAAGACGTTTTAGGGTTTCCGTTACGTAAGGGAATGTATTGTTCCTA
ATATGGTATTTAGTGCCACCCCGATCAATCACTGCCATAATTATAAGGAGTAGGATTAAATGTGAGCACATGGCAGGCCCTTTACCGAC
GTCTTCAATATTTAGCGACAGTATGGCATTTTTTTTCATGAAGCTGAATAAAAACTCTAAAGCGTACCATGTCCAAGCAGTATCATCCG
AAAGGAAAGATTGTTTGTATGCCCACTTCTAAATGAACAACGTATCTATTGCACACTTTCAAGTTGCGGATTTCAAAATTTCCGTACTT
ACCCAAAGATCTGAAAGGGCTGAACTCTAACTGGATTCTTTTAAAAACAACCTTTACTGGTCCTTTATAGGCAACTCCGAGGTATTGGA
ACACACAGATTTCGACGTCCGCAGTCAATCCTTCCTAAGTCATGATTTTCAATTCATAATTACTAGAGGTACTAGAGCATTGAATCAGA
GTGAGATCTTCTAGAGGCTACATCCGTACGTGGTAAATTAAAGAGAAGCGATTAAATGGATTTACCCATAACCGGTGAGAAGAAAGCAT

TCCACAAGTTGCTCACTACGTATTCAAGACATATGTCTACAAAGCCTTTGTGAGAATGAACTGGTCCTCAATGGAGTATTGCTTTAACC
TTTCAAAAGTGAAAAGAAACCTCTGGCTGTTGAAACATCGTTGGTTGGTTGAGTTTAATTATGCTTTAAGACACAAAGCATAGAGAAAA
TCTTCAATATACATGAAGGCAATATGCCCTAAGAAAACCAACGAGGGCAAAATCAGGCTATAGAATATCACTGTTTCTTAGGGCGTACA
CATGTTAAATTGGGAGTTTACTCACAACATTCTCAAGATCAATTAGATTTAACAAAATCTGCCCTGGAATTTGTTCGTGACAAATATCC
AAACATCTCACAAGATTTCCAATACTGGAGGGGTCAATTAGGACGTTACGGTTTAACAGGTGAAGGTCAAACGGTCCAAATGGGTACAT
TATCTGAAGGACAACGTTCGCGTGTTGTTTTTGCATTGCTAGCCCTTGAGCAGCCCAATGTTCTACTACTGGATGAACCTACTAACGGT
CTGGACATTCCAACTATTGATTCTTTGGCTGAAGCTATCCTTGAATTTAACGGTGGTGTTGTTGTCGTATCTCACGATTTCAGATTGCT
GGATAAAATTGCAAAGGATATTTTTGTTGTTGAACACAAAACTGCTACAAGATGGGATGGTAGTATTTTACAATACAAGAACAAACTTG
CCAAGAATGTTGTGTTGTGATTTTAACATATTTCACAGGTTTCTCTTCACTTTTCTCAATACAACTTGTATAATCTAATTTCATTTTCT
TACATTTCTATTCTTGATATCAGATACATATTTCTTGATAAAGTGATACATAGAGATTGAGCGTAGATGCCGGATCTTTTGCTCAGGAG
CACTATTAGAAAGCTCAAAAAAA
[matches with DBVPG6765 chr XV from 1,067,200 to 1,069,547]
[matches with *ARR* genes cluster from 1,483 to 1,575]
